# Hidden order revealed by light-driven Kerr rotation in centrosymmetric bulk WSe_2_

**DOI:** 10.1038/s41699-025-00606-9

**Published:** 2025-10-29

**Authors:** Emmanuele Cappelluti, Habib Rostami, Federico Cilento

**Affiliations:** 1 Istituto di Struttura della Materia, CNR (CNR-ISM), Trieste, Italy; 2 Department of Physics, University of Bath, Claverton Down, United Kingdom; 3 Elettra-Sincrotrone Trieste S.C.p.A., Basovizza, Italy

**Keywords:** Electronic properties and materials, Two-dimensional materials, Electronic structure, Ultrafast photonics

## Abstract

Single-layer semiconducting transition-metal dichalcogenides, lacking point inversion symmetry, provide an efficient platform for valleytronics, where the electronic, orbital, magnetic, valley, and lattice degrees of freedom can be selectively manipulated by using polarized light. This task is, however, thought to be impeded in parent bulk compounds where the point inversion symmetry is restored. Exploiting the underlying quantum physics in bulk materials is thus one of the biggest paradigmatic challenges. Here we show that a sizable optical Kerr rotation can be efficiently generated without breaking point-inversion symmetry in a wide energy range on ultrafast timescales in bulk WSe_2_, by means of circularly-polarized light. We rationalize this finding as a result of the hidden spin/layer/orbital/valley order. The spectral analysis reveals distinct A-, B-, and C-exciton features, which we show to stem from a selective Pauli blocking effect on top of the hidden-order pseudospin order and of the spin Berry curvature. The Kerr response lifetime (*τ* ~ 500 fs), common to all the peaks, suggests that excitonic dynamics dominate over single-particle decay. The present report demonstrates that the hidden order at play in bulk centrosymmetric layered materials can stem out in macroscopical bulk features, opening the way for an effective exploitation of bulk WSe_2_ in novel optoelectronic and orbitronics applications.

## Introduction

Semiconducting members of group-VI transition metal dichalcogenides (TMDs) stand out as highly promising materials for the future of nanotechnology and quantum information^1–4^. These layered materials exhibit a wealth of unique physical properties when manipulated at the single-layer level. Due to the bipartite honeycomb lattice structure and the lack of inversion symmetry, single-layer TMDs introduce a novel quantum number − the valley index − which is intricately entangled with charge, spin, layer, and orbital degrees of freedom. The valence bands at the ± K valleys feature eigenstates with opposite chiral *d*-orbital structures, $\vert {d}_{{x}^{2}-{y}^{2}}\rangle\pm i\vert {d}_{xy}\rangle$, inducing a finite quantum-geometric (Berry) curvature with opposite signs in each valley, revealing a nontrivial topological character^1,5^. The chiral nature of the valence bands makes each valley uniquely sensitive to light polarization, governed by valley-dependent selection rules^1,2,5^. These features make TMDs an ideal platform for *valleytronics*^6^ and *orbitronics*^7,8^- allowing selective targeting of specific valleys and atomic orbitals with custom optical probes^9^. The valley-dependent selection rules have been extensively studied in single-layer TMDs using tools such as photoluminescence^10–16^, transport^17^, angle-resolved photoemission spectroscopy (ARPES)^18^, and optical and magneto-optical probes^19^, also in the time-domain^2,20–28^. A key feature of these techniques is the pump-driven dichroism, where probe signals differ between opposite circular polarizations of the pump.

In contrast, bulk TMDs are often seen as less efficient for manipulating entangled quantum degrees of freedom, due to the AB stacking in the 2H structure, which restores inversion symmetry and forbids valley polarization under time-reversal symmetry. Within this context, valley polarization is thus usually achieved by applying an external out-of-plane electric/magnetic field, thus breaking the inversion symmetry^29–34^. Accordingly, an optical dichroism (different optical response for opposite circular light polarization) is usually tuned by the strength of the external field. Recent research has shown that *hidden pseudospin* orders^35^—the entangled *local* order absent at the *global* scale—can be exploited without breaking the point-inversion symmetry in centrosymmetric bulk materials to leverage the functional properties of individual layers in a multilayer system under equilibrium conditions^31,35^. This underlying hidden order has been primarily investigated in TMDs through ARPES, focusing on the spin polarization of the excited photoelectron density ejected from the valence bands. Spin-dependent dichroism in the ARPES signal disappears on very short timescales, *t* ~ 50-100 fs^36–40^.

Here we show that the optical response of centrosymmetric bulk WSe_2_ can be efficiently manipulated, without breaking point-inversion symmetry, across a wide energy range (*ℏω* ≈ 1.4–2.8 eV) and over a long timescale (~ 0.5 ps) using a circularly polarized ultrashort pump pulse at the A-exciton frequency. This manipulation is revealed by a pronounced transient optical Kerr rotation, with distinct features (and opposite signs) at the A-, B-, and C-exciton energies. Unlike single-layer TMDs, where Kerr rotation is typically observed with nearly degenerate pump-probe setups at A-exciton resonance, our results are robustly explained by a hidden order between spin, valley, layer, and orbital degrees of freedom, governed by optical selection rules and Berry curvature. Through microscopic quantum field analysis, we replicate the experimental results and uncover the origin and relative signs of these features across different excitonic ranges. The relatively long Kerr lifetime suggests that electron-hole excitations in bulk WSe_2_ persist far longer than previously indicated by ARPES. This work opens new possibilities for efficiently manipulating quantum degrees of freedom in bulk TMDs, bypassing the limitations of single-layer systems.

## Results

**Fig. 1:**
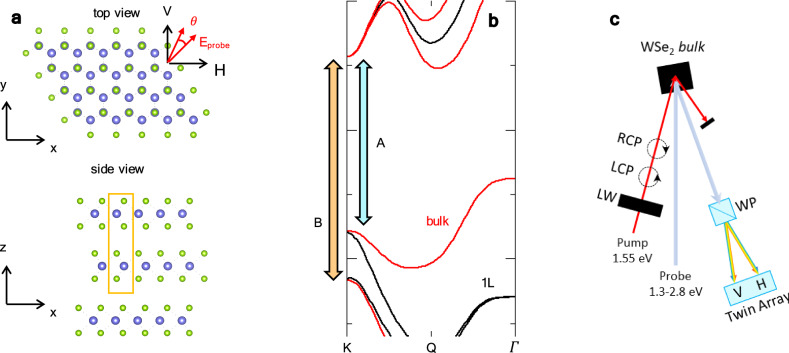
Crystal structure, band structure and geometry of the time-resolved experiment. **a** Crystal structure of the bulk WSe_2_ crystal, from a top view (top panel) and a side view (bottom panel). Tungsten (selenium) atoms are indicated in blue (green). The orange box denotes the bulk unit cell including two WSe_2_ layers. Also shown here is the 45^∘^ polarization of the incoming probe photon, whereas *θ* indicates the rotation angle of the polarization of the reflected probe beam. **b** Schematic band-structure of bulk TMDs (red lines), compared with the band structure of single-layer (black lines). The vertical arrow represents the particle-hole optical transitions responsible for the A- and B-exciton resonances. **c** Sketch of the experimental configuration: the spectra of the horizontal (H) and vertical (V) components of a broadband supercontinuum beam (1.3 − 2.8 eV), split and dispersed by a Wollaston polarizer (WP), are detected by a twin array detector. The polarization of the pump beam at *ℏω*_pump_ = 1.55 eV is switched from right-circular (RCP) to left-circular (LCP) by means of a liquid crystal variable waveplate (LW). The probe impinges the sample at near-normal incidence, while the pump impinges at an angle of ≈ 20^∘^. The bulk WSe_2_ crystal is aligned as in panel (**a**), with its *x*-axis along the horizontal direction.

The bulk semiconducting TMDs with chemical formula *MX*_2_ (*M* = Mo, W; *X* = S, Se) share a 2H lattice structure where the unit cell contains two stacked layers *MX*_2_ with a relative rotation of 180 degrees (Fig. 1a). It is known that interlayer couplings in bulk TMDs changes many of these compounds from direct-to indirect-bandgap semiconductors, with an indirect gap between the *Γ* and Q points of the Brillouin zone (Fig. 1b)^41,42^. Despite of this, however, the dominant optical features of bulk systems stem from particle-hole excitations close to the ± K points, just as for single-layer compounds, and are characterized by two exciton features, termed A and B, which are related, respectively, to the particle-hole excitations between the spin-split upper and lower valence bands and the nearly-degenerate conduction band^41^. Typical values of A and B exciton resonances in bulk WSe_2_ at room (low) temperature are *E*_A_ ~ 1.6(1.7) eV, *E*_B_ ~ 2.2(2.1) eV, respectively, slightly larger than in single-layer compounds^43–45^. Like other TMDs, a further high-energy broad shoulder, usually denoted as “C-exciton”, is reported in WSe_2_, which is often associated in literature with band-nesting conditions occurring along the *Γ*-K path of the Brillouin zone^13^.

In our setup^46^, in order to reveal in an efficient way the possible optical Kerr rotation, we perform an experiment (see sketch in Fig. 1c) where a polarization-resolved broadband probe, covering the photon energy range 1.3 − 2.8 eV, is used to detect the pump-induced ultrafast rotation of the optical tensor of a bulk WSe_2_ crystal, in a similar way as a Kerr rotation detected in monolayer TMDs under magnetic fields. The WSe_2_ crystal is oriented as sketched in Fig. 1a, and the probe incoming electric field is fixed oriented at 45 degrees in the *x*-*y* plane. The polarization of the outgoing reflectivity is hence detected simultaneously along the horizontal (H) zigzag direction and along the vertical (V) armchair direction. Due to such a geometrical configuration, the reflectivity along the H- and V-directions is identical under equilibrium conditions. As a pump pulse, we use the laser fundamental at *ℏω*_pump_ = 1.55 eV, which is nearly resonant to the first A-exciton state. The pump polarization can be quickly switched between left-circular (LCP) and right-circular (RCP) by means of a liquid-crystal variable waveplate. The incident fluence was set to 500 *μ*J/cm^2^. Δ*R*/*R* denotes the normalized differential reflectivity, measured as a function of the pump-probe delay *t* and the probe photon energy *ℏω*.

**Fig. 2:**
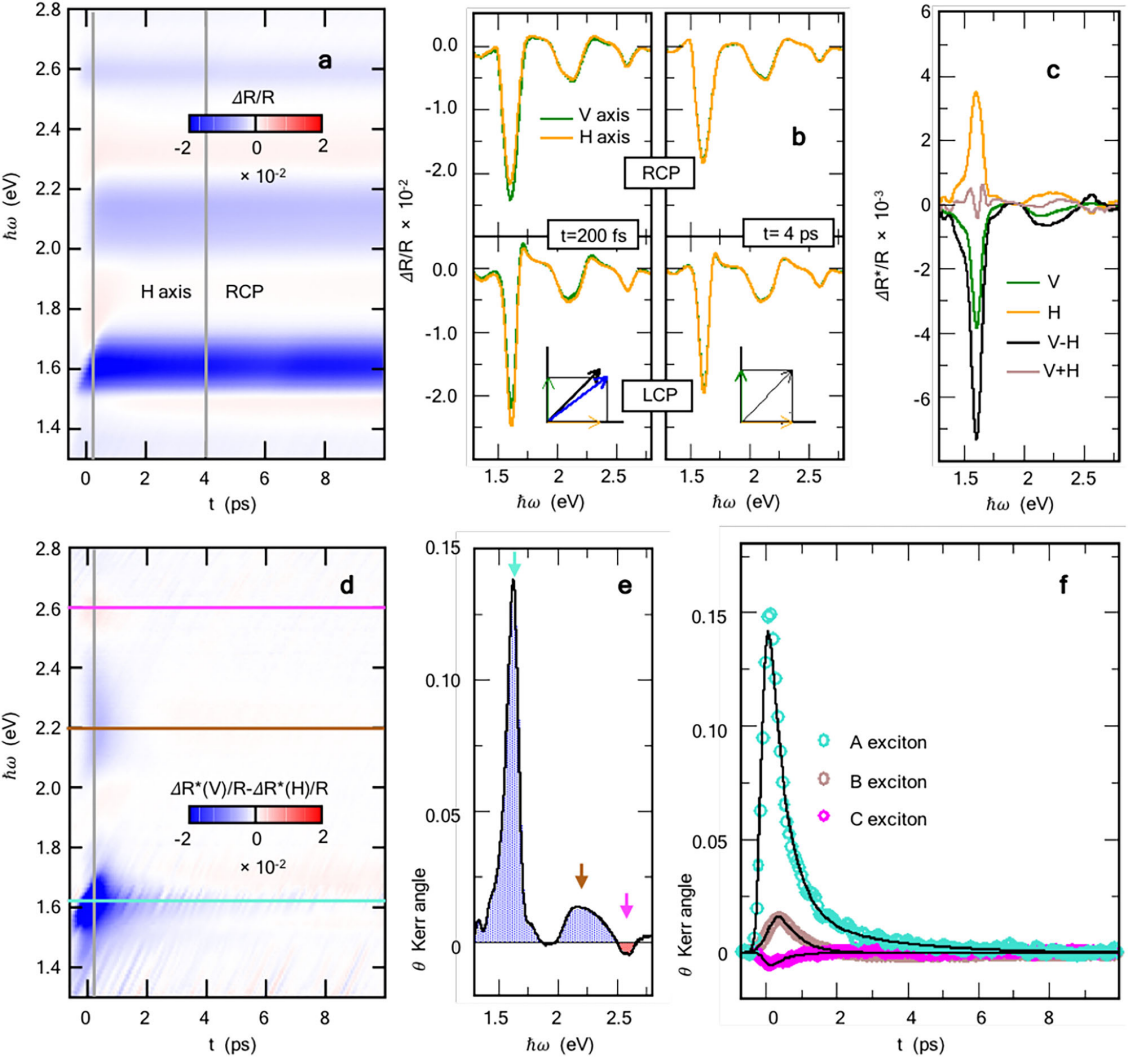
Ultrafast pump-driven Kerr rotation in bulk WSe_2_. **a** Exemplary Δ*R*/*R* map for the H probe component, acquired with RCP pump polarization (the other combinations are reported in Sec. I of the Supplementary Information), showing typical spectral features for the A, B and C excitons, at energies *ℏω* ≈ 1.6, 2.2, and 2.6 eV. The vertical grey lines at *t* = 200 fs and *t* = 4 ps mark the delays at which the spectral analysis, shown in (**b)** has been performed. (**b**) Spectral profiles of Δ*R*/*R* for the different combinations of pump and probe polarizations at *t* = 200 fs and *t* = 4 ps. The insets shows a sketch of the corresponding rotation of the reflected beam polarization. (**c**) Dichroism profiles ΔR^*^/*R* extracted from **b** at *t* = 200 fs along the V and H directions. Also shown are their sum and difference. In particular, the difference signal ΔR^*^/*R*(*V*) − ΔR^*^/*R*(*H*) is proportional to the optical Kerr rotation angle. The negligible signal ΔR^*^/*R*(*V*) + ΔR^*^/*R*(*H*) provides a check for the validity of the present analysis. **d** Energy vs. time color map of the optical dichroism ΔR^*^/*R*(*V*) − ΔR^*^/*R*(*H*), which is proportional to the rotation angle of the reflected polarization, triggered upon switching the helicity of the pump pulse polarization; **e** Net Kerr rotation angle *θ* as a function of the probe photon energy *ℏω* at *t* = 200 fs (vertical grey line in (**d**); **f** Ultrafast dynamics of the Kerr angle *θ* at the three representative features (colored symbols corresponding to horizontal cuts in (**d**). Also shown are the corresponding fits (black solid lines).

Figure 2 a shows one of the four Δ*R*/*R* maps acquired simultaneously, specifically for H probe polarization and RCP pump polarization (the other maps are reported in Fig. 1 of the Supplementary Information). Such spectra are sensitive to the broad-frequency effects of the total pump-driven photo-excitations in both valleys from the valence to the conduction bands. Sharp features with negative Δ*R*/*R* at *ℏω* = Δ_A_ ≈ 1.6 eV and *ℏω* = Δ_B_ ≈ 2.0 − 2.2 eV signal the excitonic states A and B, respectively^47^. Here we note an overall predominance of negative Δ*R*/*R*, pointing out that such spectral features are governed by a true modification of the optical intensity rather than by an energy shift due to the screening modulation. A clear feature is also detected at *ℏω* = Δ_C_ ≈ 2.6 eV, at the energy corresponding to the C exciton in bulk WSe_2_^43^. The onset of these features has been rationalized in single-layer TMDs in terms of the combined effect of Pauli blocking and of pump-driven bandgap renormalization^47^. The time scale of the relaxation dynamics of all these features is typically slow (~ 34 − 44 ps, depending on which feature), consistent with the previous literature^20,22,25,47–49^, and reflects the fact that the energy gap imposes a bottleneck to the recombination of electrons and holes.

In order to extract the appropriate Kerr signal, we analyze the normalized differential reflectivity Δ*R*/*R* for different circular polarizations of the pump photon and for different H/V orientations. The Δ*R*/*R* spectra for these four configurations are shown in Fig. 2b for time delay *t* = 200 fs and *t* = 4 ps, corresponding to vertical grey lines in Fig. 2a. At *t* = 4 ps the normalized differential reflectivity Δ*R*/*R* does not display any sensitivity to the polarization of the RCP / LCP pump. The observed optical profile of Δ*R*/*R* in this regime can thus be attributed to bandgap renormalization induced by pump-modified electronic screening. We note that Δ*R*/*R* has a slightly different profile along the H/V probe directions. This fact is likely due to a small finite incidence angle (≲ 4^∘^) of the probe beam that makes the two polarization states slightly inequivalent. For each given H or V direction, a significant dependence on the circular pump polarization is reported on the other hand, at *t* = 200 fs, especially close to each of the three excitonic energies. This is mostly evident close to the A-exciton resonance at *ℏω* = 1.6 eV, where in the V direction, RCP gives rise to larger ∣Δ*R*/*R*∣ than LCP. Polarization-induced changes in Δ*R*/*R* are reversed along the H direction, with RCP leading in that case to a smaller ∣Δ*R*/*R*∣ at *ℏω* = 1.6 eV than LCP, with a reasonably good symmetry. A similar picture is found to be valid in the energy ranges *ℏω* ≈ 2.2 eV and *ℏω* ≈ 2.6 eV, corresponding to the B and C excitons, although the magnitude of the effect in this case is strongly reduced. This reversible sensitivity to the circular polarization of the pump cannot be attributed to a pure change of electronic screening.

On the phenomenological ground, the present observations can be recast in terms of a modified optical conductivity tensor ***σ***, with 2 × 2 components in the *x*-*y* space^50,51^. In the absence of pumping (or with a long delay, e.g., *t* = 4 ps), ***σ*** is purely diagonal with *σ*_*xx*_ = *σ*_*yy*_. The observed pump-driven changes in Δ*R*/*R* can thus be rationalized in terms of a pump-driven onset of an off-diagonal term with *σ*_*xy*_ = − *σ*_*yx*_ ≠ 0, giving rise to a net (Kerr) rotation of the linear probe polarization soon after the photo-excitation, as sketched in the insets of Fig. 2b. The Kerr nature of the observed dichroism is evident in Fig. 2c where we plot the dichroism of Δ*R*/*R* [ΔR^*^/*R* = Δ*R*/*R*_RCP_ − Δ*R*/*R*_LCP_] at *t* = 200 fs along the V and H directions. We note that the energy-shape of ΔR^*^/*R* along the H probe direction is nicely matched by a corresponding opposite ΔR^*^/*R* along the V direction. Such analysis is assessed at a quantitative level in the same figure where we further show the sum, ΔR^*^/*R*(*V*) + ΔR^*^/*R*(*H*), and the difference, ΔR^*^/*R*(*V*) − ΔR^*^/*R*(*H*), between the two axes. While the latter quantity can be directly interpreted as a pump-induced Kerr signal, the first one represents what *it is not* the Kerr effect. Its negligibility with respect to the Kerr signal, ∣ΔR^*^/*R*(*V*) + ΔR^*^/*R*(*H*)∣ ≪ ∣ΔR^*^/*R*(*V*) − ΔR^*^/*R*(*H*)∣, provides thus a robust validation of our results. Equipped with such a spectral analysis for bulk WSe_2_, we plot in Fig. 2d the energy-time dependence of the optical dichroism ΔR^*^/*R*(*V*) − ΔR^*^/*R*(*H*), which is proportional to the Kerr angle. For comparison, we performed a similar analysis for bulk WS_2_ and MoTe_2_ at the same pump energy *ℏω*_pump_ ≈ 1.55 eV (which for these materials is not resonant with the A-exciton), and no trace of any Kerr optical response was found in the whole energy range investigated (see Figs. 2 and 3 in the Supplementary Information).

Three important features are worth being pointed out in the data for bulk WSe_2_: (*i*) we show evidence of a sizable optical rotation at *all* the three excitonic frequencies *ℏω* ≈ 1.6, 2.2, 2.6 eV, well above the pumping energy *ℏω*_pump_ ≈ 1.55 eV, pointing out that pumping at the A-exciton resonance has a profound impact on the entangled optical properties of all these features in a wide frequency range; (*ii*) we observe the same sign for the optical dichroism (i.e., Kerr rotation) for both the A and B exciton spectra, but an *opposite* sign for the C-exciton. This can be better visualized in Fig. 2e where we plot the Kerr angle *θ*(*t*, *ℏω*) at *t* = 200 fs (vertical line cut in Fig. 2d) as obtained by using the procedure outlined in Ref. ^46^ (Methods). We characterize each Kerr feature with the energies *ℏω*_*i*_ = 1.63, 2.2, and 2.6 eV, where its maximum $| {\theta }^{\max }(t=200\,\,\text{fs}\,,\omega )|$ occurs. The ratio of the corresponding intensities at *ℏω*_*i*_ (*i* = A, B, C) is found $| {\theta }_{{\rm{B}}}^{\max }| /| {\theta }_{{\rm{A}}}^{\max }| \approx 10 \%$, $| {\theta }_{{\rm{C}}}^{\max }| /| {\theta }_{{\rm{A}}}^{\max }| \approx 3.4 \%$. As we will see later, the relative sign among these features can be rationalized and reproduced by means of a microscopical quantum-field analysis, revealing the role of the valley/spin/layer hidden order and of the interlayer coupling; (*iii*) although the exciton population dynamics is found to decay with a long timescale *τ*_long_ ~ 30 − 40 ps (see Fig. 2a), the optical Kerr rotation at the relevant energies *ℏω*_*i*_ displays much shorter, but still sizable lifetimes. The time evolution of *θ*(*t*, *ℏω*_*i*_) at the three characteristic energies [horizontal cuts in panel (**d**)] is shown in Fig. 2f. In order to assess the characteristic timescales of the three features, we fit each dataset with a standard fitting procedure, including the pump-probe cross-correlation driven risetime and one or two exponential decays (see the Methods). The fit curves are superimposed as black solid lines over the experimental points in Fig. 2f. We find that the time dynamics of the Kerr angle *θ*(*t*, *ℏω*) at the A-exciton energy *ℏω* = 1.63 eV cannot be described by a single exponential timescale, whereas a bi-exponential decay ${\theta }_{{\rm{A}}}(t)\propto {a}_{1}\exp [-t/{\tau }_{{\rm{A,fast}}}]+{a}_{2}\exp [-t/{\tau }_{{\rm{A,slow}}}]$ provides a good agreement, with a large component *a*_1_ characterized by a fast dynamics *τ*_A,fast_ ≈ 476 ± 30 fs, followed by a weaker component (*a*_2_/*a*_1_ ≈ 0.12) with a slower dynamics *τ*_A,slow_ ≈ 2315 ± 50 fs. The analysis of the time dynamics at the B, C exciton energies displays similar fast timescales with *τ*_B_ ≈ 528 ± 30 fs and *τ*_C_ ≈ 511 ± 30 fs, while no slower component for these features is detected.

## Discussion

### Optical Kerr rotation in bulk samples from quantum hidden order

The optical Kerr rotation upon circularly polarized pumping implies a time-reversal-symmetry breaking, and it has been theoretically predicted and observed in monolayer TMDs^2,19–25,27,28,52^ where it has been attributed to the lack of a center of symmetry, together with the valley-selective population induced by the circularly polarized pumping. To our knowledge, the present observation is the first direct report of a net Kerr effect in *bulk* TMDs while preserving the crystal point-inversion symmetry. The natural questions are then: how a Kerr rotation is activated by circularly-polarized pumping in bulk TMDs without breaking the point-inversion-symmetry? Why similar spectral fingerprints can be found in both (non-centrosymmetric) monolayer and (centrosymmetric) bulk TMDs?

To address these issues, we performed a quantum-field analysis (see Section II of Supplementary Information for technical details) of the optical response in the presence of circularly polarized photoinduced particle-hole excitations resonant with the A-exciton energy. In order to capture all the observed Kerr spectral features, we consider a non-interacting model that is enough to capture the relevant physics, and we employ a six-band **k** ⋅ **p** model that generalizes to the bulk case the three-band model, previously employed for single-layer TMDs^53,54^. Within this context, only relevant *d*-orbitals of the metal atoms, with atomic orbital angular momentum *l*_at_ = 0, ± 2, are retained, providing a good modeling of the relevant valence and conduction bands, as well as of a third block of bands with ∣*l*_at_∣ = 2 *d*-orbitals, associated with the high-energy excitons and which have been proposed to be responsible for the C-exciton peak. Following previous literature, we assume a local and spin-diagonal spin-orbit coupling. Such an approximation, which has been validated in Refs. ^53,55^, allows us to retain the spin as a good quantum number even in bulk centrosymmetric systems where bands are double-degenerate, obeying the Kramer’s theorem. We further expand the total Hamiltonian close to the high-symmetry points *ν*K (*ν* = ±) of the bulk Brillouin zone, so that $\hat{H}({\bf{k}})\approx {\sum }_{s,\nu }{\hat{H}}_{s,\nu }({\bf{p}})$, where ${\hat{H}}_{s,\nu }$ is defined in the 6 × 6 orbital space of bulk systems, *s* = ± 1 is the spin, *ν* = ± 1 represents the valley index ± K, and **p** = **k** − *ν*K is the relative momentum with respect to the corresponding valley.

Before discussing the full spectral properties of the Kerr response, it is worth clarifying the underlying mechanisms giving rise to a finite Kerr rotation. To this purpose, we focus for the moment on the static limit of the off-diagonal optical tensor *σ*_*xy*_(*ℏω* → 0), where the Kerr response extrapolates smoothly to the Hall rotation. Within this framework, it is known that the term *σ*_*xy*_(0) can be conveniently related to the underlying topological properties encoded in the Berry curvature *Ω*_*n*,*s*_(**k**), i.e., *σ*_*xy*_ = (*e*^2^/*ℏ*)∑_*n*,*s*,*ν*_∑_**p**_*Ω*_*n*,*s*,*ν*_(**p**)*f*[*E*_*n*,*s*,*ν*_(**p**)], where *n* is the band index and *f*[*x*] the momentum/band population factor that, at equilibrium, corresponds to the thermal Fermi-Dirac distribution. We neglect for the moment the high-energy conduction bands, which play a role only for high-energy spectral features, and we consider the reduced 4 × 4 Hamiltonian where only the low-energy conduction and valence bands are retained, with an effective interlayer hopping term which mainly couples the valence bands. Such a Hamiltonian has been previously used to underline the intrinsic entanglement between the valley, the layer and the spin polarization^31^, which can conveniently be defined in the orbital/layer space. This approach, however, cannot be generalized in a straightforward way to the Hall/Kerr response since the Berry curvature is defined in the band-space.

**Fig. 3:**
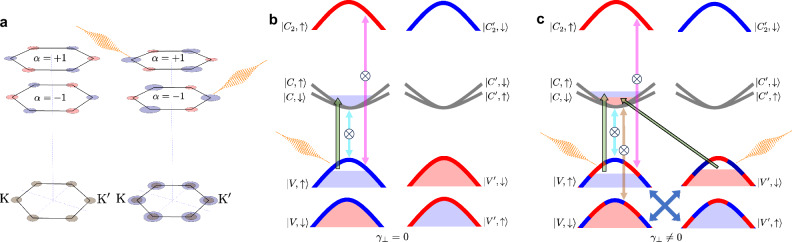
Quantum hidden order and effects of selection rules on the Kerr optical response. **a** Schematic representation of the different contributions to the *σ*_*xy*,*α*,*ν*_ Kerr optical rotation resolved for the layer and valleys. For the sake of representation, the spin degree has been averaged. The bottom line represents the total layer-averaged *σ*_*xy*,*ν*_ = ∑_*α*_*σ*_*xy*,*α*,*ν*_. Blue/red areas represent states resulting in a positive/negative Kerr angle, respectively, while grey areas stand for null Kerr rotation. The left panel shows the hidden order before pumping. LCP laser pumping in the right panel photo-excites charges at K in layer *α* = + 1, and at -K in layer *α* = − 1. In both cases, it results in a net increase of *σ*_*xy*_ > 0 in each valley, resulting in a finite Kerr rotation. The system still obeys inversion symmetry, and each valley is equivalent. **b** Representative selection rules and Pauli-blocked optical transitions at *ν* = 1 valley in the absence of interlayer coupling. The absorption of an LCP photon tuned at the A-energy triggers particle-hole transitions (upward green arrow) only from the *E*_V,*↑*_ state with ∣*l*_at_∣ = 2 (blue bands) to *E*_C,*↑*_ with *l*_at_ = 0 (grey bands). The resulting photo-excited charges *n*_V,*↑*_ = *n*_C,*↑*_ ≠ 0 induce Pauli blocking (crossed colored vertical double-arrows) at both the A- and C-resonance energies. **c** Same as (**b**) in the presence of a finite interlayer coupling, which leads to a mixed *l*_at_ character for the *E*_V,*s*_ bands, resulting in a large charge transfer from *E*_V,*↑*_ to *E*_C,*↑*_, but also in a finite charge transfer from *E*_V,*↓*_ to *E*_C,*↓*_, hence *n*_V,*↓*_ = *n*_C,*↓*_ ≠ 0. A Pauli-blocking is consequently active also for the particle-hole transitions responsible for the B-resonance.

Despite this apparent limitation, in the following, we show that in bulk TMDs it is possible to define in a compelling way a layer-projection of the Berry curvature for each band. This task is made feasible in bulk TMDs due to the layered electronic structure *and* to the specific form of the interlayer coupling, which is negligible for the conduction bands. Within this context, using the above Hamiltonian ${\hat{H}}_{s,\nu }({\bf{p}})$, we can thus evaluate a layer/spin/valley-resolved Berry curvature *Ω*_*n*,*s*,*ν*,*α*_(**p**), which allows us to compute as well a layer/spin/valley-resolved off-diagonal response *σ*_*xy*,*s*,*ν*,*α*_, with *α* = ± 1 standing for the layer index. Under equilibrium conditions, we obtain at the valley points **p** = 0:

1 $${\sigma }_{xy,s,\nu ,\alpha }^{{\rm{eq}}}=-\frac{{e}^{2}}{\hslash }\left[\tilde{\lambda }\left({\Omega }_{{\rm{A}}}-{\Omega }_{{\rm{B}}}\right)s+\left({\Omega }_{{\rm{A}}}+{\Omega }_{{\rm{B}}}\right)\nu \alpha \right],$$

where *Ω*_A_ is the Berry curvature of the top valence band at the valley point (associated with the A-exciton optical transitions), *Ω*_B_ is the Berry curvature of the bottom valence band (associated with the B-exciton optical transitions), and $\tilde{\lambda }=\lambda /\sqrt{{\lambda }^{2}+{\gamma }_{\perp }^{2}}$ is a dimensionless parameter expressing the relative relevance of the spin-orbit coupling *λ* with respect to the interlayer coupling *γ*_⊥_. Eq. (1) displays the layer/spin/valley-resolved hidden-order of the Kerr/Hall response. The first term in Eq. (1) descends directly from the analysis of the finite spin-resolved Berry curvature, without the need of a layer-projection, and it reflects the possible onset of a finite Hall/Kerr rotation upon breaking of the time-reversal symmetry^52^. Obeying the space-inversion symmetry, it does not depend on the valley index *ν*. The second term in Eq. (1) is on the other hand a novel feature which appears only within a layer-resolved analysis and it reveals that in bulk TMDs at equilibrium, although a net (layer-averaged) valley-Hall (valley-Kerr) response is null, a hidden order is present, with a finite valley/layer Hall conductivity response ${\sigma }_{xy,\nu ,\alpha }^{{\rm{eq}}}\,\ne 0$ as a consequence of the underlying entanglement between valley *ν* and layer *α*. A representative plot of the hidden order of ${\sigma }_{xy,\nu ,\alpha }^{{\rm{eq}}}$ is shown in Fig. 3a, showing a staggered pattern in the layer/valley space. Such layer/valley entanglement plays a crucial role in our context since it points out an alternative path for generating a finite Hall/Kerr response in bulk TMDs, not related to a direct time-reversal-symmetry breaking, but as a consequence of a hidden *inversion-symmetry breaking*.

To understand how circularly-polarized pumping can exploit such hidden order in inducing a finite Kerr rotation, we compute at the valley points (**p** = 0) the optical selection rules, which can be related to the Berry curvature through the valuable expression^56^:

2 $$\begin{array}{lll}{{\mathcal{P}}}_{n\to m,s,\nu }^{\zeta }\,=\,{\left\vert {J}_{x,s,\nu }^{nm}(0)\right\vert }^{2}/2+{\left\vert {J}_{y,s,\nu }^{nm}(0)\right\vert }^{2}/4\\\qquad\qquad\quad -\zeta {\left[{E}_{n,s,\nu }(0)-{E}_{m,s,\nu }(0)\right]}^{2}{\Omega }_{nm,s,\nu }(0)/4.\end{array}$$

Eq. (2) is proportional to the probability of absorbing a photon with chiral polarization *ζ* = ± accompanied by a particle-hole excitation from the *n*-band to the *m*-band with spin *s* in the valley *ν*. On the grounds of the above discussion, and as detailed in Section II of Supplementary Information, we recognize that the layer indices do not mix in Eq. (2) so that we can properly define *layer-resolved* selection rules ${{\mathcal{P}}}_{n\to m,s,\nu ,\alpha }^{\zeta }$. Joining ${{\mathcal{P}}}_{n\to m,s,\nu ,\alpha }^{\zeta }$ with the detailed expressions of the layer/spin/valley-resolved Berry curvature *Ω*_*n*,*s*,*ν*,*α*_, we can finally derive the total effect on the off-diagonal optical response of the absorption of a circularly-polarized photon tuned at the A-exciton edge:

3 $$\delta {\bar{\sigma }}_{xy,s,\nu ,\alpha }^{\zeta }=\delta {\sigma }_{xy}^{{\rm{spin}}}s+\delta {\sigma }_{xy}^{{\rm{orb}}}\nu \alpha +\delta {\sigma }_{xy}^{{\rm{dichro}}}\zeta +\delta {\sigma }_{xy}^{{\rm{full}}}\zeta s\nu \alpha .$$

The first term in (3) is the only one that survives after averaging over all the internal degrees of freedom, and it is the one responsible for the Kerr response observed in our experimental setup. It arises as consequence of two different channels: the effect of the selection rules of a chiral photon on the spin sector, ∝ *ζs*, which acts on top of the spin structure of the hidden order ∝ *s* [first term in Eq. (1)]; and the effect of the selection rules induced by a chiral photon on the orbital order, ∝ *ζνα*, exploiting the underlying hidden order ∝ *να* [second term in Eq. (1)]. A schematic picture of the onset of a finite Hall/Kerr response from such entanglement is shown in the right panel of Fig. 3a: a circularly-polarized photon will act at the *ν* = + 1 valley mainly on layer *α* = + 1 and in similar way at *ν* = − 1 on layer *α* = − 1. The net result is an off-diagonal term with a well-defined sign *δσ*_*xy*_ > 0 in both valleys, obeying thus the lattice inversion symmetry. The third and fourth terms in (3), on the other hand, describe the effects of the pumping that do not give rise to any dichroism, as related, for instance, to the change of the overall charge distribution. They obey the same symmetries of the bulk system, and they follow thus the same pattern of the hidden order. They can be probed in the presence of an explicit space/time breaking by external fields. Finally, the second term of (3) conveys the full entanglement among all the degrees of freedom. The above scenario is consistent with previous ARPES investigations of the hidden valley/helicity/spin order in bulk TMDs^36–40,57^.

### Spectral analysis and time dependence

After having clarified the mechanisms giving rise to a light-induced Kerr response in centrosymmetric bulk TMDs, we now investigate the fundamental information encoded in the spectral profile of the optical Kerr rotation, with a particular focus on the origin of the three main features outlined in Fig. 2e and on their relative sign. We evaluated the optical conductivity *σ*_*ij*_(*ω*) (*i*, *j* = *x*, *y*) within a non-interacting Kubo formalism using the full 6 × 6 Hamiltonian, retaining thus also the high-energy conduction bands. With the aim of focusing on the role of the selection rules dictated by the hidden order, we do not explicitly include in our modeling the many-body Coulomb interaction that would lead to a band-renormalization within a *GW* approach and to a bound exciton state within a Bethe-Salpeter scheme. It is, on the other hand, nowadays well assessed that fully interacting systems inherit, close to the valleys, the same selection rules as the non-interacting one, making the present analysis suitable to reveal the mechanisms which different optical Kerr features arise and their relative sign. Similarly as in Ref. ^54^, three main contributions can be identified, ${\sigma }_{ij}^{{\rm{A}}}(\omega )$, ${\sigma }_{ij}^{{\rm{B}}}(\omega )$, ${\sigma }_{ij}^{{\rm{C}}}(\omega )$, associated with the interband transitions responsible for the A-, B-, and C-exciton optical features, respectively. The off-diagonal part of each of these terms fulfills at a spectral level, under equilibrium conditions, the same symmetries as their static limit in Eq. (1), and it vanishes in the absence of symmetry breaking or of external fields.

Finite spectral features *δσ*_*xy*_(*ω*) appear however, upon the effect of circularly-polarized pumping tuned at the A-exciton resonance. For standard fluence values, pump-driven particle hole excitations are localized very close to valleys **p** = 0, modifying in a non-thermal way the population factors *f*[*E*_*n*_] ≈ *f*^eq^[*E*_*n*_] + *δf*[*E*_*n*_]. The selection rules reported in Eq. (2) provide useful guidance for evaluating such effects. More in details, we obtain:

4 $${{\mathcal{P}}}_{{\rm{V}}\to {\rm{C}},s,\nu }^{\zeta }(0)=(1-\tilde{\lambda }\zeta s-\nu \zeta +\tilde{\lambda }s\nu )/4,$$

5 $${{\mathcal{P}}}_{{\rm{V}}\to {{\rm{C}}}^{{\prime} },s,\nu }^{\zeta }(0)=(1-\tilde{\lambda }\zeta s+\nu \zeta -\tilde{\lambda }s\nu )/4,$$

where ${{\mathcal{P}}}_{{\rm{V}}\to {\rm{C}}}^{\zeta }$, ${{\mathcal{P}}}_{{\rm{V}}\to {{\rm{C}}}^{{\prime} }}^{\zeta }$ describe the probability of transition from the top valence band V to the (degenerate) low-energy conduction bands C, C${}^{{\prime} }$. Using such information within the Kubo computation of the off-diagonal optical response, we can estimate the integrated spectral area ${I}_{{\rm{K}}}^{i}={\sum }_{s,\nu }\int{\rm{Im}}\,\,\delta {\sigma }_{xy,s,\nu }^{i}(\omega )d\omega$, for each of the optical Kerr features:

6 $${I}_{{\rm{K}}}^{{\rm{A}}}\propto -\frac{{v}_{1}^{2}}{{\Delta }_{{\rm{A}}}}(1+3{\tilde{\lambda }}^{2})\zeta ,$$

7 $${I}_{{\rm{K}}}^{{\rm{B}}}\propto -\frac{{v}_{1}^{2}}{{\Delta }_{{\rm{B}}}}(1-{\tilde{\lambda }}^{2})\zeta ,$$

8 $${I}_{{\rm{K}}}^{{\rm{C}}}\propto \frac{{v}_{3}^{2}}{{\Delta }_{{\rm{C}}}}\tilde{\lambda }(1+\tilde{\lambda })\zeta ,$$

where the factors *v*_1_, *v*_3_ are related to the current matrix element between different bands.

Noticeable information is encoded in Eqs. (6)–(8): (*i*) they predict that the Kerr rotation at the A- and B-resonances have the same sign, and an opposite sign with respect to the Kerr rotation at the C-edge, in perfect agreement with the experimental observations; (*ii*) the onset of a Kerr response at the B-resonance in bulk TMDs under circularly-polarized pumping is intimately related to the interlayer coupling, and it would vanish in the decoupled-layer limit $\tilde{\lambda }\to 1$; (*iii*) on the other hand, the spin-orbit coupling has a fundamental role in the Kerr feature at the C-edge, and it would disappear in the opposite limit of no spin-orbit (or extremely large interlayer coupling) $\tilde{\lambda }\to 0$. On the quantitative level, taking from the literature the values of $\tilde{\lambda }=0.959$,^31^ and the ratio *v*_3_/*v*_1_ = 0.733,^54^ we estimate $| {I}_{{\rm{K}}}^{{\rm{A}}}| > | {I}_{{\rm{K}}}^{{\rm{B}}}| > | {I}_{{\rm{K}}}^{{\rm{C}}}|$, with $| {I}_{{\rm{K}}}^{{\rm{B}}}| /| {I}_{{\rm{K}}}^{{\rm{A}}}| \approx 0.24$ and $| {I}_{{\rm{K}}}^{{\rm{C}}}| /| {I}_{{\rm{K}}}^{{\rm{A}}}| \approx 0.16$. Note that such estimates are based on a non-interacting analysis and do not take into account thus many-body renormalization and other effects). More precise first-principle analyses, using *GW* or Bethe-Salpeter-based calculations, predict, for instance, a much smaller ratio of the optical oscillation strengths ${v}_{3}^{2}/{v}_{1}^{2}\approx 0.04$^15^, in even further agreement with the experimental observation.

We can achieve a more microscopic insight into the processes responsible for each spectral feature, and on their time-dynamics, by relating, using the Kubo formalism, the Kerr spectral intensity at different energies to pump-driven photo-excited charge densities *n*_*n*,*s*_. In particular, focusing at a single valley *ν* = 1 (in bulk systems, both valley are equivalent) and for left-circularly-polarized photons, we get:

9 $${I}_{{\rm{K}}}^{{\rm{A}}}(t)\propto \frac{{v}_{1}^{2}}{{\Delta }_{{\rm{A}}}}\left[{c}_{\lambda }^{2}({n}_{{\rm{C}},\uparrow }-{n}_{{{\rm{C}}}^{{\prime} },\downarrow })-{s}_{\lambda }^{2}({n}_{{{\rm{C}}}^{{\prime} },\uparrow }-{n}_{{\rm{C}},\downarrow })+({c}_{\lambda }^{2}-{s}_{\lambda }^{2})\left({n}_{{\rm{V}},\uparrow }-{n}_{{\rm{V}},\downarrow }\right)\right],$$

10 $${I}_{{\rm{K}}}^{{\rm{B}}}(t)\propto \frac{{v}_{1}^{2}}{{\Delta }_{{\rm{B}}}}\left[{s}_{\lambda }^{2}({n}_{{\rm{C}},\uparrow }-{n}_{{{\rm{C}}}^{{\prime} },\downarrow })+{c}_{\lambda }^{2}({n}_{{\rm{C}},\downarrow }-{n}_{{{\rm{C}}}^{{\prime} },\uparrow })\right],$$

11 $${I}_{{\rm{K}}}^{{\rm{C}}}(t)\propto -\frac{{v}_{3}^{2}}{{\Delta }_{{\rm{C}}}}{c}_{\lambda }^{2}({n}_{{\rm{V}},\uparrow }-{n}_{{\rm{V}},\downarrow }).$$

Here the prefactors ${c}_{\lambda }^{2}=(1+\tilde{\lambda })/2$ and ${s}_{\lambda }^{2}=(1-\tilde{\lambda })/2$ take into account the (time-independent) magnitude of the optical activities for given interband transitions, whereas the population *n*_*n*,*s*_(*t*) can have a time dynamics.

Let us first consider as a template case the limit of decoupled layers *γ*_⊥_ = 0 in the bulk 2H structure. In that case ${c}_{\lambda }^{2}=1$, ${s}_{\lambda }^{2}=0$. Keeping in mind that the two layers of the unit cell are rotated each other by 180^∘^, the electronic structure of the bulk systems is thus obtained by the degenerate superposition of the electronic states *ψ*_*n*_(**k**, *α* = + 1) and of the electronic states *ψ*_*n*_(**k**, *α* = − 1). In this limit the bulk bands are thus completely layer-localized, with bands of layer *α* = + 1 corresponding to the K point of the single-layer system, and bands of layer *α* = − 1 corresponding to K${}^{{\prime} }$. At *t* = 0 the absorption of a LCP photon with energy tuned at the A-exciton edge *ℏω* ≈ Δ_A_ triggers at *ν* = 1 particle-hole transitions only in layer *α* = + 1 (Fig. 3b). Considering also the role of the geometrical phase winding of the Bloch states *l*_geo_^11,58,59^, particle-hole transitions are allowed only from the valence state *E*_V,*↑*_, with atomic orbital angular momentum *l*_at_ = 2 (blue bands) to the conduction state *E*_C,*↑*_ with atomic orbital angular momentum *l*_at_ = 0 (grey bands)^11^, with a net total angular momentum change Δ*l*_tot_ = − 1. The only finite photo-excited charge densities will be thus completely spin-polarized *n*_C,*↑*_ = *n*_V,*↑*_ ≠ 0. On the other hand, no particle-hole transitions are allowed from the valence state *E*_V,*↓*_ to the conduction band ${E}_{{{\rm{C}}}^{{\prime} },\downarrow }$, although fulfilling the energy-resonant requirement, because *E*_V,*↓*_ is localized on the layer *α* = − 1 and it borrows from these in-layer states the main orbital angular momentum *l*_at_ = − 2 (red bands) which is not coupled with the absorption of an LCP photon. The concomitant Pauli blocking of the conduction and valence bands, *E*_C,*↑*_, *E*_V,*↑*_ with a change of the selected orbital angular momentum Δ*l*_tot_ = − 1, gives rise to the finite Kerr response at the A-edge energy, with an overall spectral area as in Eq. (9).

However, the photoexcited hole density in the valence band *E*_V,*↑*_ is also responsible for the Pauli blocking of the optical transitions between the valence band *E*_V,*↑*_ and the high-energy conduction band ${E}_{{{\rm{C}}}_{2},\uparrow }$, associated with the C-exciton shoulder. However, due to the different orbital characteristics of conduction bands C_2_ (*l*_at_ = 2) with respect to conduction bands C (*l*_at_ = 0), such Pauli blocking involves a change of total angular momentum Δ*l*_tot_ = 1 *opposite* to the one involved in the A-resonance, and hence a Kerr effect at *ℏω* = Δ_C_ with *opposite sign* than at *ℏω* = Δ_A_. The very experimental observation of a Kerr response at the C-edge, furthermore with the correct sign as predicted by the theory, supports thus the identification of the broad C-exciton feature as induced by a strong band nesting for optical transitions between the valence and the high-energy conduction bands at the valley points.

It is worth noticing here that, within the assumption of no interlayer coupling, no Kerr response at the B-resonance is expected as a direct effect of circularly-polarized pumping. A finite Kerr rotation at the B-exciton edge upon circularly polarized pumping has been reported in single-layer TMDs, where it was explained as an effect of the valley depolarization induced by intervalley scattering^20,24,25^. We rule out this possible explanation of our experimental observation, as it will imply an *opposite* sign of the Kerr angle at *ℏω* ≈ Δ_B_ with respect to *ℏω* ≈ Δ_A_. Our theoretical analysis [Eq. (7)] shows, however, that a finite Kerr response at the B-resonance, with the correct sign as experimentally observed, is a direct product of the circularly polarized pumping, even in the absence of many-body scattering processes. The physics describing this effect is mathematically captured in Eq. (10) and is schematically summarized in Fig. 3c. The fundamental feature to be highlighted here is that the interlayer coupling hybridizes states in different layers with opposite orbital angular momentum. The mixed orbital-angular-momentum character is depicted in Fig. 3c as dashed blue-red bands. The remarkable aspect is that at the valley *ν* = + 1, the photo-induced particle-hole excitations, and the corresponding photo-charges, can occur not only between *E*_V,*↑*_ and *E*_C,*↑*_ (thick vertical green arrow), but also, to a minor extent, between *E*_V,*↓*_ and *E*_C,*↓*_ (tiny oblique green arrow). The corresponding photo-induced charges at *t* = 0 can be estimated as ${n}_{{\rm{C}},\uparrow }={c}_{\lambda }^{2}$, ${n}_{{\rm{C}},\downarrow }={s}_{\lambda }^{2}$, ${n}_{{\rm{V}},\uparrow }={c}_{\lambda }^{2}$, ${n}_{{\rm{V}},\downarrow }={s}_{\lambda }^{2}$, whereas ${n}_{{{\rm{C}}}^{{\prime} },\uparrow }={n}_{{{\rm{C}}}^{{\prime} },\downarrow }=0$. The Kerr response at the B-resonance is thus induced by the two parallel channels: Pauli blocking the ${E}_{{{\rm{V}}}^{{\prime} },\uparrow }\leftrightarrow {E}_{{\rm{C}},\uparrow }$ transitions, driven by the large conduction charge *n*_C,*↑*_, but with a small current matrix element ${s}_{\lambda }^{2}$ induced by the interlayer coupling; and the Pauli blocking of the ${E}_{{{\rm{V}}}^{{\prime} },\downarrow }\leftrightarrow {E}_{{\rm{C}},\downarrow }$ transitions, driven by the small conduction charge *n*_C,*↓*_, but with a large (mainly intralayer) current matrix element ${s}_{\lambda }^{2}$. Both these channels trigger an angular momentum change Δ*l*_tot_ = − 1, just at the processes associated with the A-resonance, and induce thus a Kerr response at the B-edge with the same sign as at the A-resonance.

Equipped with Eqs. (9)–(11), having identified the origin and the physical processes for all three spectral features in the Kerr response, we comment on the observed time-dependence. In order to determine the entanglement between the different degrees of freedom and the optical selection rules, Eqs. (9)–(11) [and (4), (5)] have been derived using a non-interacting model. Such features are not expected to change where the many-body interactions, and the actual formation of bound excitons are taken into account, with the obvious warning that exciton resonance energies should be considered as inferred from the experiments, including thus the many-body band renormalization and the exciton binding energy. Eqs. (9)–(11) thus provide a useful guidance to rationalize the charge time-dynamics, including the effective time-dependence *n*_*n*,*s*_(*t*). Starting from the pump-induced populations at *t* = 0, ${n}_{{\rm{C}},\uparrow }(0)={n}_{{\rm{V}},\uparrow }(0)={c}_{\lambda }^{2}$, ${n}_{{\rm{C}},\downarrow }={n}_{{\rm{V}},\downarrow }={s}_{\lambda }^{2}$, ${n}_{{{\rm{C}}}^{{\prime} },\uparrow }(0)={n}_{{{\rm{C}}}^{{\prime} },\downarrow }(0)=0$, the overall Kerr response fades out either when spin-flip/layer-flip processes lead to equal spin/layer populations at the valley points (e.g., *n*_V,*↑*_ ≈ *n*_V,*↓*_, ${n}_{{\rm{C}},\uparrow }\approx {n}_{{{\rm{C}}}^{{\prime} },\uparrow }$); or when, in bulk TMDs, photoexcited charges migrate to other points of the Brillouin zone, forming, for instance, an exciton. Eqs. (9)–(11) appear thus very powerful since the analysis of the time-dependence of the Kerr response at the A-, B- and C-edges allows in principle to trace in an independent way the time-dynamics of the conduction (${I}_{{\rm{K}}}^{{\rm{B}}}$) and valence (${I}_{{\rm{K}}}^{{\rm{C}}}$) charges, as well as their joint effect (${I}_{{\rm{K}}}^{{\rm{A}}}$). On this ground, it is strikingly remarkable that all three Kerr features display a similar time-decay with *τ*_fast_ ≈ 500 fs (leaving aside the weak long-lived component of the A feature). This observation strongly suggests thus that our set-up, with energy pumping tuned at the A-resonance, does *not* induce free itinerant charges *n*_V,*s*_, *n*_C,*s*_, ${n}_{{{\rm{C}}}^{{\prime} },s}$ in an independent way in the conduction and valence bands, but it simply affects the total number of available excitons. Photo-induced conduction and valence charges are thus still bound together, with the time-scale *t* ≲ 500 fs, by the exciton binding energy, and they obey thus the same time dynamics, hampering also the independent migration towards different points of the Brillouin zone.

The common decay time that we observe for all three Kerr features at room temperature is comparable with the reported time-dynamics of the Kerr A-feature in monolayer WSe_2_ at room temperature^22,25,60^, suggesting thus that the observed time-dynamics reveals the time decay due to the spin-flip/layer-flip processes, which are similar as in monolayer systems (note that due to the 2H stacking, layer-flip processes in bulk TMDs are similar to inter-valley processes in monolayer compounds). Time-resolved angle-resolved photoemission spectroscopy (tr-ARPES) explores, on the other hand, a different physics, tracing the dynamics of the (unbound) charges left by the photoemission process (in the valence bands or in the pump-populated conduction bands). Not bound in the exciton pairs, these charges can optimize their energy by easily migrating to their own minima in bulk samples (Q-point for conduction, *Γ*-point for valence bands), with a much faster dynamics^37,39,40,54,60^. Our results thus present additional information, and they are thus not incompatible with tr-ARPES data in bulk WSe_2_, which report a much shorter time decay (*τ*_B,ARPES_ ≈ 100 fs) for the dichroism in the conduction bands^37,39,40^.

In conclusion, we have reported evidence of a large Kerr optical rotation, driven by circularly polarized light, in a wide energy range on bulk WSe_2_. The present results show that efficient optical manipulation is affordable not only in single-layer TMDs with broken inversion symmetry, but also in centrosymmetric bulk WSe_2_, circumventing the limitation of atomically thin samples. We rationalize the presence of a pump-induced Kerr effect in centrosymmetric bulk TMDs as a result of the time-reversal-symmetry-breaking induced by the circularly polarized light absorption, exploiting the hidden quantum order between the orbital content, valleys, and layers. This scenario allows for a finite Kerr rotation in bulk samples without a selective valley population. We also argue that the joint analysis of the spectral intensities of the three Kerr features provides a powerful insight on the dynamics of the photoexcited particle-hole excitations. On this ground, our findings suggest that bound excitons can live for a rather long timescale (*τ* ≈ 500 fs) also in bulk TMDs. Similar dichroism and Kerr effects could be exploited in the bulk structure of other transition metal dichalcogenides, such as Mo-based compounds. Furthermore, the known presence of a large spin-orbit coupling in WSe_2_ implies that a sizable spin polarization is also possible in bulk TMDs, in agreement thus with the findings of Ref. ^40^.

## Methods

### Samples and experiment details

High-quality bulk single-crystals of 2H-WSe_2_ were purchased from HQ Graphene. Their in-plane orientation was determined by ex-situ LEED (Low Energy Electron Diffraction) experiments, and the samples were mounted in the optical setup accordingly to this information. TR-OS experiments were performed using the experimental setup described in^46^. Briefly, the output of a Ti:Sapphire regenerative Amplifier (Coherent RegA), delivering 50 fs pulses at 800 nm (1.55 eV) and 250 kHz, is split for obtaining pump and probe pulses. A broadband supercontinuum pulse extending in a range 400-1100 nm and linearly-polarized at 45 degrees with respect to the optical table is obtained by focusing 1 *μ*J/pulse of energy (≈ 250 mW of power) in a 3 mm thick Sapphire window. The reflected beam from the sample is separated in its horizontal (H) and vertical (V) polarization components and spectrally dispersed by a Wollaston Polarizer, and finally detected by a pair of 512-pixels CMOS linear array detectors by Hamamatsu. The pump pulse at 800 nm is polarized circularly with a liquid crystal waveplate from Thorlabs, which allows to quickly (≈ 50 ms) switch between the two circular polarization states. The spot size on the sample amounts to 160 *μ*m and 50 *μ*m full width at half maximum (FWHM) for the pump and the probe, respectively. The two beams cross at an angle of 20 degrees.

### Evaluation of the Kerr angle from reflectivity anisotropy

We describe here the procedure employed to estimate the Kerr rotation angle from the measurement of the reflectivity anisotropy upon circularly polarized pumping.

For this task, we essentially followed the scheme nicely described in Refs. ^51,61^.

Using the quantum field theory, we first computed microscopically, as described in the previous Section, the optical conductivity tensor

12 $$\hat{\sigma }(\omega )=\left(\begin{array}{cc}{\sigma }_{xx}(\omega )&{\sigma }_{xy}(\omega )\\ -{\sigma }_{xy}(\omega )&{\sigma }_{xx}(\omega )\end{array}\right),$$

where we have assumed *σ*_*yy*_(*ω*) = *σ*_*xx*_(*ω*), and hence the complex dielectric function $\hat{\epsilon }(\omega )$ through the standard relation $\hat{\epsilon }(\omega )={\epsilon }_{\infty }\hat{I}+4\pi i{\sigma }_{xx}(\omega )/\omega$.

In our setup, we shed incoming light at 45 degrees in the *x*-*y* plane, thus with the same components *E*_*x*_ = *E*_*y*_. The optical response along the along *x* (H) and *y* (V) is governed thus by:

13 $${\epsilon }_{{\rm{V}}}(\omega )={\epsilon }_{xx}(\omega )+{\epsilon }_{xy}(\omega ),$$

14 $${\epsilon }_{{\rm{H}}}(\omega )={\epsilon }_{xx}(\omega )-{\epsilon }_{xy}(\omega ).$$

The corresponding reflectivity along the two axes was thus determined as:

15 $${r}_{\alpha }(\omega )=\frac{1-\sqrt{{\epsilon }_{\alpha }(\omega )}}{1+\sqrt{{\epsilon }_{\alpha }(\omega )}},$$

and the absolute value of the reflectivity as:

16 $${R}_{\alpha }(\omega )={\left\vert {r}_{\alpha }(\omega )\right\vert }^{2},$$

where *α* = H, V.

The Kerr angle *θ*(*ω*) has been eventually computed through the standard formula^46^:

17 $$\theta (\omega )=\frac{1}{2}\frac{{R}_{{\rm{H}}}(\omega )-{R}_{{\rm{V}}}(\omega )}{{R}_{{\rm{H}}}(\omega )+{R}_{{\rm{V}}}(\omega )}.$$

### Time-dependence fitting procedure

The time-traces have been fitted using the conventional approach, making use of one or two exponential decays of the form ${a}_{i}\exp (-t/{t}_{i})$, *i* = 1, 2, where *a*_*i*_ is the amplitude of the exponential component, *t*_*i*_ its the corresponding time-constant, and *t* represents the pump-probe delay. The data have been fitted using Igor Pro and performing the convolution of the above expression with a Gaussian function accounting for the finite temporal resolution of the setup, due to the pump-probe cross correlation. The Gaussian FWHM used is 300 fs, which is mostly determined by the large pump-probe crossing angle.

## Supplementary information

Supplementary Information

## Data Availability

The data and code that support the plots within this paper and other findings of this study are available from the corresponding authors upon reasonable request.
