# Supplementary material for: Hidden order revealed by light-driven Kerr rotation in centrosymmetric bulk WSe2
Source: NPJ 2D Mater Appl. 2025 Oct 29;9(1):89. doi: 10.1038/s41699-025-00606-9 (PMC12576942; doi:10.1038/s41699-025-00606-9)
Supplement: Supplementary file 1 — Supplementary Information [file 41699_2025_606_MOESM1_ESM.pdf]

Supplementary Information for: Hidden Order Revealed by Light-Driven Kerr Rotation in Centrosymmetric Bulk WSe_2

Emmanuele Cappelluti,[1,*] Habib Rostami,[2,†] and Federico Cilento[3,‡]

[1] Istituto di Struttura della Materia, CNR (CNR-ISM), 34149 Trieste, Italy
[2] Department of Physics, University of Bath, Claverton Down, Bath BA2 7AY, United Kingdom
[3] Elettra-Sincrotrone Trieste S.C.p.A., 34149 Basovizza, Italy

I. NORMALIZED DIFFERENTIAL REFLECTIVITY MAPS

Here we present the differential reflectivity maps $\Delta R/R$ as a function of time t and probe frequency $\hbar\omega$ under different setups, switching the left- vs right circular polarization of the pump, and collecting the horizontal (H) vs. vertical (V) probe reflectivity components. All the four possible combinations for bulk WSe_2, with a pump energy tuned at the A-exciton energy $\hbar\omega_{\rm pump} = 1.55$ eV are shown in Fig. 1. In Figs. 2-3 we show also the corresponding maps for bulk WS_2 and WTe_2, respectively, with fixed pump energy at $\hbar\omega_{\rm pump} = 1.55$ eV, far thus from resonant pump conditions. No dichroism, and no Kerr effect is observed under these conditions.

II. OPTICAL SELECTION RULES AND HIDDEN ORDER

For a detailed description of the relevant features of bulk TMDs, we generalized for bulk TMDs the three-band $\mathbf{k}\cdot\mathbf{p}$ model, previously employed for single-layer TMDs[1,2]. Within this context only the relevant d-orbitals of the metal atoms with atomic orbital angular momentum $l_{\rm at} = 0, \pm 2$ are retained, providing a good modeling of the low-energy conduction bands $E_0(\mathbf{p})$ with dominant $l = 0$ character; of the valence bands $E_{\rm B}(\mathbf{p})$, which correspond actually at the bottom block of bands with dominant $|l_{\rm at}| = 2$ character; and of a block of high-energy conduction bands, $E_{\rm T}(\mathbf{p})$, with dominant $|l_{\rm at}| = 2$ character, which are responsible for higher energy optical transitions[2–4]. Taking into account that the two MX_2 layers of the bulk unit cell have a relative rotation of 180 degrees, a suitable Hilbert space at the valley ν was provided by $\psi^\dagger_{s,\nu} = (\psi^\dagger_{s,\nu,1}, \psi^\dagger_{s,\nu,-1})$ where

$$\psi^\dagger_{s,\nu,\alpha} = (d^\dagger_{0,s,\alpha}, d^\dagger_{{\rm B},s,\nu,\alpha}, d^\dagger_{{\rm T},s,\nu,\alpha}). \quad (1)$$

Here $d^\dagger_{0,s,\alpha}$ creates in layer α an electron with spin s in the orbital $d_{3z^2-r^2}$; $d^\dagger_{{\rm B},s,\nu,\alpha}$ creates in layer α an electron with spin s and with chiral orbital linear combination $d_{x^2-y^2} + i\nu d_{xy}$; $d^\dagger_{{\rm T},s,\nu,\alpha}$ creates in layer α an electron with spin s and with chiral orbital linear combination $d_{x^2-y^2} - i\nu d_{xy}$. In this basis the bulk Hamiltonian reads:

$$\hat{H}_{s,\nu}(\mathbf{p}) = \begin{pmatrix} \hat{h}_{s,\nu}(\mathbf{p}) & \hat{h}_\perp \\ \hat{h}^\dagger_\perp & \hat{h}_{s,-\nu}(\mathbf{p}) \end{pmatrix}, \quad (2)$$

where $\mathbf{p} = \mathbf{k} - \nu K$ is the relative momentum with respect to the valley, and $\hat{h}_{s,\nu}(\mathbf{p})$ is the $\mathbf{k}\cdot\mathbf{p}$ Hamiltonian for the single-layer[2]:

$$\hat{h}_{s,\nu}(\mathbf{p}) = \begin{pmatrix} E_0(p) & \hbar v_1 p_{\nu,-} & \hbar v_2 p_{\nu,+} \\ \hbar v_1 p_{\nu,+} & E_{\rm B}(p) + \lambda s\nu & \hbar v_3 p_{\nu,-} \\ \hbar v_2 p_{\nu,-} & \hbar v_3 p_{\nu,+} & E_{\rm T}(p) - \lambda s\nu \end{pmatrix}, \quad (3)$$

with $p_{\nu,\pm} = \nu p_x \pm i p_y$ and λ the spin-orbit coupling. The only (spin-diagonal) interlayer term in $\hat{h}_\perp$ is known to be the coupling $\gamma_\perp$ between the block of $E_{\rm B}$ bands. Other interlayer couplings mix E_0 and $E_{\rm T}$ with the large energy difference[5], and their effect can be neglected. The properties of the bulk system were conveniently investigated in the rotated basis that diagonalizes Eq. (2) at $\mathbf{p} = 0$,

$$\phi^\dagger_{s,\nu} = (d^\dagger_{{\rm C},s,\nu}, d^\dagger_{{\rm V},s,\nu} d^\dagger_{{\rm C}_2,s,\nu}, d^\dagger_{{\rm C}',s,\nu}, d^\dagger_{{\rm V}',s,\nu}, d^\dagger_{{\rm C}'_2,s,\nu},), \quad (4)$$

where

$$d_{\mathrm{C},s,\nu} = d_{0,s,1}, \tag{5}$$
$$d_{\mathrm{C}',s,\nu} = d_{0,s,-1}, \tag{6}$$
$$d_{\mathrm{V},s,\nu} = \cos\chi_{s,\nu} d_{\mathrm{B},s,\nu,1} + \sin\chi_{s,\nu} d_{\mathrm{B},s,-\nu,-1}, \tag{7}$$
$$d_{\mathrm{V}',s,\nu} = -\sin\chi_{s,\nu} d_{\mathrm{B},s,\nu,1} + \cos\chi_{s,\nu} d_{\mathrm{B},s,-\nu,-1}, \tag{8}$$
$$d_{\mathrm{C}_2,s,\nu} = d_{\mathrm{T},s,\nu,1}, \tag{9}$$
$$d_{\mathrm{C}_2',s,\nu} = d_{\mathrm{T},s,-\nu,-1}. \tag{10}$$

Here $\chi_{s,\nu} = (1/2)\arccos(\tilde{\lambda}\nu s)$, where we defined $\tilde{\lambda} = \lambda/\sqrt{\lambda^2+\gamma_\perp^2}$. For sake of compactness, we define also $c_{s,\nu} = \cos\chi_{s,\nu}$ and $s_{s,\nu} = \sin\chi_{s,\nu}$. In the basis (4) the Hamiltonian reads:

$$\hat{\mathcal{H}}_{s,\nu}(\mathbf{p}) = \begin{pmatrix} \hat{h}_{s,\nu}(\mathbf{p}) & \hat{h}_\perp \\ \hat{h}_\perp^\dagger & \hat{h}'_{s,\nu}(\mathbf{p}) \end{pmatrix}, \tag{11}$$

where

$$\hat{h}_{s,\nu}(\mathbf{p}) = \begin{pmatrix} E_{\mathrm{C},s,\nu}(p) & \hbar v_1 c_{s,\nu} p_{\nu,-} & \hbar v_2 p_{\nu,+} \\ \hbar v_1 c_{s,\nu} p_{\nu,+} & E_{\mathrm{V},s,\nu}(p) & \hbar v_3 c_{s,\nu} p_{\nu,-} \\ \hbar v_2 p_{\nu,-} & \hbar v_3 c_{s,\nu} p_{\nu,+} & E_{\mathrm{C}_2,s,\nu}(p) \end{pmatrix}, \tag{12}$$

$$\hat{h}'_{s,\nu}(\mathbf{p}) = \begin{pmatrix} E_{\mathrm{C}',s,\nu}(p) & \hbar v_1 c_{s,\nu} p_{-\nu,-} & \hbar v_2 p_{-\nu,+} \\ \hbar v_1 c_{s,\nu} p_{-\nu,+} & E_{\mathrm{V}',s,\nu}(p) & \hbar v_3 c_{s,\nu} p_{-\nu,-} \\ \hbar v_2 p_{-\nu,-} & \hbar v_3 c_{s,\nu} p_{-\nu,+} & E_{\mathrm{C}_2',s,\nu}(p) \end{pmatrix}, \tag{13}$$

and

$$\hat{h}_\perp = \begin{pmatrix} 0 & \hbar v_1 s_{s,\nu} p_{-\nu,+} & 0 \\ \hbar v_1 s_{s,\nu} p_{-\nu,+} & 0 & \hbar v_3 s_{s,\nu} p_{-\nu,-} \\ 0 & \hbar v_3 s_{s,\nu} p_{-\nu,-} & 0 \end{pmatrix}. \tag{14}$$

The current operators $\hat{J}_{i,s,\nu}(\mathbf{p}) = \partial\hat{\mathcal{H}}_{s,\nu}(\mathbf{p})/\partial p_i$, can be also promptly derived in the band basis from Eq. (11).

The Hamiltonian in Eq. (11) defines at $\mathbf{p}=0$ the two split valence bands, $E_{\mathrm{V},s,\nu} = E_\mathrm{B} + \sqrt{\lambda^2+\gamma_\perp^2}$, $E_{\mathrm{V}',s,\nu}(0) = E_\mathrm{B} - \sqrt{\lambda^2+\gamma_\perp^2}$, with mixed-layer character. They correspond to the antibonding and bonding interlayer states, respectively. On the other hand, due to the negligible interlayer coupling, the states of the low-energy and high-energy conduction bands, are strongly localized on each layer, in accordance with Eqs. (5)-(10), with $E_{\mathrm{C},s,\nu} = E_{\mathrm{C}',s,\nu} = E_0$ being essentially degenerate, while $E_{\mathrm{C}_2,s,\nu}$ $E_{\mathrm{C}',s,\nu}$ display a sizable spin-orbit splitting, $E_{\mathrm{C}_2,s,\nu} = E_\mathrm{T} - \lambda s\nu$, $E_{\mathrm{C}_2',s,\nu} = E_\mathrm{T} + \lambda s\nu$. The energy differences: $\Delta_\mathrm{A} = E_0 - E_\mathrm{V}$, $\Delta_\mathrm{B} = E_0 - E_{\mathrm{V}'} =$, $\Delta_\mathrm{C} = E_\mathrm{T} - \lambda - E_\mathrm{V}$, define the edges, in the non-interacting limit, for the particle-hole continuum associated with the A- B- and C-exciton resonances, respectively.

A. Layer-resolved selection rules and layer/spin/valley/photon hidden order in bulk TMDs

In order to analyze the topological character and the optical selection rules of bulk TMDs we focused on the 4×4 reduced relevant Hilbert space where only the valence and low-energy conduction bands were retained[6–8], neglecting at this stage the additional high-energy conduction bands.

The Berry curvature for each band was computed as $\Omega_{n,s,\nu}(\mathbf{p}) = \sum_{m\neq n}\Omega_{nm,s,\nu}(\mathbf{p})$, where[7]

$$\Omega_{nm,s,\nu}(\mathbf{p}) = -2\mathrm{Im}\left[\frac{\langle J_{x,s,\nu}(\mathbf{p})\rangle_{n,m}\langle J_{y,s,\nu}(\mathbf{p})\rangle_{m,n}}{[E_{n,s,\nu}(\mathbf{p}) - E_{m,s,\nu}(\mathbf{p})]^2}\right]. \tag{15}$$

Here $\langle J_i\rangle_{n,m}$ is the matrix element of the expectation value of the current operators $\hat{J}_i$ expressed in the band-basis (4), and we have implicitly used the fact that the Hamiltonian (11) is diagonal in both the spin and valley degrees of freedom.

For small pump fluences, the light-induced particle-hole excitations are localized nearby the valley points $\mathbf{p}=0$, so that the physical properties are well captured by an analysis at $\mathbf{p}=0$. We got in particular:

$$\Omega_{\mathrm{C},s,\nu}(0) = (\Omega_\mathrm{A}+\Omega_\mathrm{B})\,\nu + \tilde{\lambda}\,(\Omega_\mathrm{A}-\Omega_\mathrm{B})\,s, \tag{16}$$
$$\Omega_{\mathrm{C}',s,\nu}(0) = -\,(\Omega_\mathrm{A}+\Omega_\mathrm{B})\,\nu + \tilde{\lambda}\,(\Omega_\mathrm{A}-\Omega_\mathrm{B})\,s, \tag{17}$$
$$\Omega_{\mathrm{V},s,\nu}(0) = -2\tilde{\lambda}\Omega_\mathrm{A} s, \tag{18}$$
$$\Omega_{\mathrm{V}',s,\nu}(0) = 2\tilde{\lambda}\Omega_\mathrm{B} s. \tag{19}$$

where $\Omega_\mathrm{A} = \hbar^2 v^2/\Delta_\mathrm{A}^2$ is the Berry curvature associated with the particle-hole transitions responsible for the A-exciton edge, and $\Omega_\mathrm{B} = \hbar^2 v^2/\Delta_\mathrm{B}^2$ is the Berry curvature associated with the particle-hole transitions responsible for the B-exciton edge.

Within the same framework, we derive the optical selection rules governing the absorption of a photon with chiral polarization $\zeta = \pm$ driving a particle-hole excitation from the n-band to the m-band[9]:

$$\mathscr{P}^\zeta_{n\to m,s,\nu} = \left|J^{nm}_{x,s,\nu}(0)\right|^2/2 + \left|J^{nm}_{y,s,\nu}(0)\right|^2/4 - \zeta\,[E_{n,s,\nu}(0) - E_{m,s,\nu}(0)]^2\,\Omega_{nm,s,\nu}(0)/4. \tag{20}$$

In the specific case of a pump energy tuned at the A-exciton edge, i.e. accompanied by a particle-hole excitation from the valence band V to the conduction band C or C′, we get the dimensionless expressions:

$$\mathscr{P}^\zeta_{\mathrm{V}\to\mathrm{C},s,\nu}(0) = (1 - \tilde{\lambda}\zeta s - \nu\zeta + \tilde{\lambda} s\nu)/4, \tag{21}$$

$$\mathscr{P}^\zeta_{\mathrm{V}\to\mathrm{C}',s,\nu}(0) = (1 - \tilde{\lambda}\zeta s + \nu\zeta - \tilde{\lambda} s\nu)/4. \tag{22}$$

Further insight was gained by splitting the current operators in their layer-projected components, $\hat{J}_{i,s,\nu} = \sum_\alpha \hat{J}_{i,s,\nu,\alpha}$. It should be remarked that, in general, this projection is *not* sufficient for defining *layer-projected* Berry curvatures and *layer-projected* selection rules, since different layer components can mix in Eq. (15). A careful analysis showed however that at $\mathbf{p} = 0$ such mixing does not occur in our case thanks to the layered structure of bulk TMDs and to the reduced effect of the interlayer coupling. We could thus introduce in a compelling way a *layer-resolved* Berry curvature $\Omega_{n,s,\nu,\alpha}(0)$ such that $\Omega_{n,s,\nu}(0) = \sum_\alpha \Omega_{n,s,\nu,\alpha}(0)$. The possibility of defining a layer-resolved Berry curvature allowed us to treat in a compelling way the layer index as an additional quantum degree of freedom and to evaluate *layer-resolved* (as well a spin/valley-resolved) topological properties, selection rules and, in an ultimate analysis, a *layer-resolved* off-diagonal optical response $\sigma_{xy,s,\nu,\alpha}$ through the relation:

$$\sigma_{xy,s,\nu,\alpha} = -\frac{e^2}{\hbar}\sum_{\mathbf{p},n}\Omega_{n,s,\nu,\alpha}(\mathbf{p}) f[E_{n,s,\nu}(\mathbf{p})], \tag{23}$$

where $f[E]$ is the population factor (non necessarily thermal). In order to investigate the entanglement between the different degrees of freedom, since the Berry curvatures are commonly peaked at the valley points, we focus in detail on the contribution at $\mathbf{p} = 0$, where $\sigma_{xy,s,\nu,\alpha} \approx -(e^2/\hbar)\sum_{\mathbf{p},n}\Omega_{n,s,\nu,\alpha}(0) f[E_{n,s,\nu}(0)]$. Using the layer-projected current operators, we obtain:

$$\Omega_{\mathrm{C},s,\nu,\alpha}(0) = \left[(\Omega_\mathrm{A} + \Omega_\mathrm{B})\,\nu + \tilde{\lambda}\,(\Omega_\mathrm{A} - \Omega_\mathrm{B})\,s\right]\frac{1+\alpha}{2}, \tag{24}$$

$$\Omega_{\mathrm{C}',s,\nu,\alpha}(0) = \left[-(\Omega_\mathrm{A} + \Omega_\mathrm{B})\,\nu + \tilde{\lambda}\,(\Omega_\mathrm{A} - \Omega_\mathrm{B})\,s\right]\frac{1-\alpha}{2}, \tag{25}$$

$$\Omega_{\mathrm{V},s,\nu,\alpha}(0) = -\Omega_\mathrm{A}\nu\alpha - \tilde{\lambda}\Omega_\mathrm{A} s, \tag{26}$$

$$\Omega_{\mathrm{V}',s,\nu,\alpha}(0) = -\Omega_\mathrm{B}\nu\alpha + \tilde{\lambda}\Omega_\mathrm{B} s. \tag{27}$$

Under equilibrium conditions we obtain thus:

$$\sigma^\mathrm{eq}_{xy,s,\nu,\alpha} = -(\Omega_\mathrm{A} + \Omega_\mathrm{B})\,\nu\alpha - \tilde{\lambda}\,(\Omega_\mathrm{A} - \Omega_\mathrm{B})\,s. \tag{28}$$

The pattern of $\sigma^\mathrm{eq}_{xy,s,\nu,\alpha}$ is depicted in Fig. 3a and it displays the intrinsic hidden order of the Kerr response including the spin, valley and layer degrees of freedom. For a bulk-sensitive spin-integrated probe, as our setup in the absence of pumping, we get $\sigma^\mathrm{eq}_{xy} = \sum_{s,\nu,\alpha}\sigma^\mathrm{eq}_{xy,s,\nu,\alpha} = 0$.

The layer-resolved Berry curvature allowed us to derive also the following layer-resolved optical selection rules:

$$\mathscr{P}^\zeta_{\mathrm{V}\to\mathrm{C},s,\nu,\alpha}(0) = (1 - \tilde{\lambda}\zeta s - \nu\zeta + \tilde{\lambda} s\nu)(1+\alpha)/8, \tag{29}$$

$$\mathscr{P}^\zeta_{\mathrm{V}\to\mathrm{C}',s,\nu,\alpha}(0) = (1 - \tilde{\lambda}\zeta s + \nu\zeta - \tilde{\lambda} s\nu)(1-\alpha)/8. \tag{30}$$

On the ground of Eqs. (29)-(30), along with Eqs. (24)-(27), we could furthermore evaluate the additional contribution $\delta\bar{\sigma}^\zeta_{xy}$ to the off-diagonal response induced by the absorption of circularly-polarized pumping. Retaining the explicit dependence of all the degrees of freedom, after a careful analysis we get:

$$\delta\sigma^\zeta_{xy,s,\nu,\alpha} \propto -\frac{2\Omega_\mathrm{A} + \Omega_\mathrm{B} + \tilde{\lambda}^2(2\Omega_\mathrm{A} - \Omega_\mathrm{B})}{4}\zeta + \frac{2\Omega_\mathrm{A} + \Omega_\mathrm{B} + \tilde{\lambda}(2\Omega_\mathrm{A} - \Omega_\mathrm{B})}{4}\nu\alpha + \frac{\tilde{\lambda}\Omega_\mathrm{A}}{2}s - \tilde{\lambda}\Omega_\mathrm{A}\zeta\alpha s\nu. \tag{31}$$

B. Optical Kerr response

In order to achieve a detailed understanding of all observed optical Kerr features, it was important to retain all the optically-active bands, as captured by the Hamiltonian (11). In similar way as for single-layer TMDs, we split thus the total response in three interband contributions[2], $\sigma_{xy} = \sigma_{xy}^{\rm v-c} + \sigma_{xy}^{\rm v-c_2} + \sigma_{xy}^{\rm c-c_2}$. The first term $\sigma_{xy}^{\rm v-c}$ accounts for interband transitions between the block of valence bands (v=V, V′) and the block of low-energy conduction bands (c=C, C′), and it is responsible for the spectral features at the A-edge and B-edge exciton energies; the second term $\sigma_{xy}^{\rm v-c_2}$ described the interband transitions between the block of valence bands (v=V, V′) and the block of high-energy conduction bands ($\rm c_2 = C_2$, $\rm C_2'$). Finally, the third term describes optical transitions between the block of low-energy conduction bands and the block of high-energy conduction bands. This term is Pauli-blocked in semiconducting bulk TMDs at equilibrium. Although these optical features can be activated upon pumping, they don't play a relevant role in the present context and we neglected them.

The term $\sigma_{xy}^{\rm v-c}$ can be further divided in a contribution arising from the V bands, as resulting in optical features at the A-exciton edge, $\sigma_{xy}^{\rm A} = \sigma_{xy}^{\rm V-c}$, and in a contribution arising from the V′ bands, as resulting in optical features at the B-exciton edge, $\sigma_{xy}^{\rm B} = \sigma_{xy}^{\rm V'-c}$. In similar way, one can isolate in $\sigma_{xy}^{\rm v-c_2}$ the contribution $\sigma_{xy}^{\rm C} = \sigma_{xy}^{\rm V-c_2}$ associated with transitions between the V bands and the band $\rm C_2$, $\rm C_2'$ with lowest energy (this latter label depends on the valley/spin index). Using Eqs. (11)-(14) and the Kubo formalism, we can thus write:

$$\sigma^{\rm A}_{xy,s,\nu}(\omega) = -i\nu \frac{e^2 v_1^2}{4\pi^2\hbar^2\omega}\Big[c^2_{s,\nu} M(E_{{\rm C},s,\nu}, E_{{\rm V},s,\nu}, \omega) - s^2_{s,\nu} M(E_{{\rm C}',s,\nu}, E_{{\rm V},s,\nu}, \omega)\Big], \tag{32}$$

$$\sigma^{\rm B}_{xy,s,\nu}(\omega) = -i\nu \frac{e^2 v_1^2}{4\pi^2\hbar^2\omega}\Big[s^2_{s,\nu} M(E_{{\rm C},s,\nu}, E_{{\rm V}',s,\nu}, \omega) - c^2_{s,\nu} M[E_{{\rm C}',s,\nu}, E_{{\rm V}',s,\nu}, \omega)\Big], \tag{33}$$

$$\sigma^{\rm C}_{xy,s,\nu}(\omega) = i\nu \frac{e^2 v_3^2}{4\pi^2\hbar^2\omega}\Big[I_{s\nu} c^2_{s,\nu} M[E_{{\rm C}_2,s,\nu}, E_{{\rm V},s,\nu}, \omega) - I_{-s\nu} s^2_{s,\nu} M[E_{{\rm C}_2',s,\nu}, E_{{\rm V},s,\nu}, \omega)\Big], \tag{34}$$

where

$$M(E_n, E_m, \omega) = \sum_{\mathbf p} \left\{ \frac{f[E_n(\mathbf p)] - f[E_m(\mathbf p)]}{E_n(\mathbf p) - E_m(\mathbf p) - \hbar\omega - i\delta} - \frac{f[E_n(\mathbf p)] - f[E_m(\mathbf p)]}{E_n(\mathbf p) - E_m(\mathbf p) + \hbar\omega + i\delta} \right\}, \tag{35}$$

and where $I_{s\nu} = (1 + s\nu)/2$ traces down that the level $E_{\rm T} - \lambda$ is associated with different bands $\rm C_2$, $\rm C_2'$ for different valley/spin indices. In semiconducting bulk TMDs at equilibrium, $f[E_{{\rm V},s,\nu}(\mathbf p)] = 1$, $f[E_{{\rm C},s,\nu}(\mathbf p)] = 0$, $f[E_{{\rm C}_2,s,\nu}(\mathbf p)] = 0$, and all the three features vanish once summed over the spin.

Finite spectral features $\delta\sigma_{xy}(\omega)$ appear however upon the effect of circularly-polarized pumping tuned at the A-exciton resonance. For standard values of fluence, pump-driven particle-hole excitation are localized very close to valleys $\mathbf p = 0$, modifying in a non-thermal way the population factors $f[E] \approx f^{\rm eq}[E] + \delta f[E]$. For energies $\hbar\omega$ close to the resonance edge, we could evaluate the change in the response functions:

$$\begin{aligned} {\rm Im}\delta M(E_1, E_2, \omega) &\approx \pi\delta[E_1(0) - E_2(0) - \hbar\omega] \sum_{\mathbf p} \{\delta f[E_1(\mathbf p)] - \delta f[E_2(\mathbf p)]\} \\ &= \pi\delta[E_1(0) - E_2(0) - \hbar\omega]\{\delta n_1 - \delta n_2\}, \end{aligned} \tag{36}$$

where δn_i is the pump-driven charge variation in the corresponding band E_i. Using the selection rules Eqs. (21)-(22) we can estimate $\delta n_{{\rm V},s,\nu} = -(1 - \tilde\lambda\zeta s)F/2$, $\delta n_{{\rm C},s,\nu} = (1 - \tilde\lambda\zeta s - \nu\zeta + \tilde\lambda s\nu)F/4$, $\delta n_{{\rm C}',s,\nu} = (1 - \tilde\lambda\zeta s + \nu\zeta - \tilde\lambda s\nu)F/4$, where F is a factor which scales linearly with the pump fluence. For all the other bands, not affected by a pumping at the A-resonance, $\delta n = 0$. With such modeling we could evaluate thus the pump-driven off-diagonal part the optical tensor, $\delta\sigma_{xy,s,\nu}(\omega)$, and the spectral integrated area of each optical Kerr feature i =A, B, C as

$$I^i_{\rm K} = \sum_{s,\nu} \int {\rm Im}\ \delta\sigma^i_{xy,s,\nu}(\omega) d\omega. \tag{37}$$

We get:

$$I^{\rm A}_{\rm K} \propto -\frac{v_1^2}{\Delta_{\rm A}}(1 + 3\tilde\lambda^2)\zeta F, \tag{38}$$

$$I^{\rm B}_{\rm K} \propto -\frac{v_1^2}{\Delta_{\rm B}}(1 - \tilde\lambda^2)\zeta F, \tag{39}$$

$$I^{\rm C}_{\rm K} \propto \frac{v_3^2}{\Delta_{\rm C}}\tilde\lambda(1 + \tilde\lambda)\zeta F. \tag{40}$$

Using the layer-resolved analysis, we computed also the different spin/layer contributions to Eqs. (38)-(40) . Focusing on $\nu = 1$, and considering for instance a left-circularly-polarized photon $\zeta = -1$, we get:

$$I^{\rm A}_{s,\alpha=1} \propto \frac{v_1^2}{\Delta_{\rm A}} \left(\frac{1+\tilde{\lambda}^2}{2} + \tilde{\lambda} s \right) F, \tag{41}$$

$$I^{\rm A}_{s,\alpha=-1} \propto -\frac{v_1^2}{\Delta_{\rm A}} \left(\frac{1-\tilde{\lambda}^2}{4} \right) F, \tag{42}$$

$$I^{\rm B}_{s,\alpha=1} \propto \frac{v_1^2}{\Delta_{\rm B}} \frac{1-\tilde{\lambda}^2}{4} F, \tag{43}$$

$$I^{\rm B}_{s,\alpha=-1} \propto 0, \tag{44}$$

$$I^{\rm C}_{s,\alpha=1} \propto -\frac{v_3^2}{\Delta_{\rm C}} \frac{(1+\tilde{\lambda})^2(1+s)}{8} F, \tag{45}$$

$$I^{\rm C}_{s,\alpha=-1} \propto \frac{v_3^2}{\Delta_{\rm C}} \frac{(1-\tilde{\lambda}^2)(1-s)}{8} F. \tag{46}$$

Few interesting properties can be noticed here: (*i*) the Kerr spectral features at the C-resonance $\hbar\omega = \Delta_{\rm C}$ arises from a unique spin sector, and they are expected to arise from fully spin-polarized transitions. On the contrary, the Kerr feature at the B-edge $\hbar\omega = \Delta_{\rm B}$ is totally spin-independent; (*ii*) the Kerr features at the B-edge stem also from a unique layer; (*iii*) in the regime of weak interlayer coupling (which is realized in many bulk TMDs) the Kerr features at the A-resonance $\hbar\omega = \Delta_{\rm A}$, as well as the C-one, are related to fully spin- and layer-polarized optical excitations. Eq. (36) allowed us to relate the Kerr spectral intensity at different energies to pump-driven photo-excited charge densities. In particular, focusing at a single valley $\nu = 1$, and for left-circularly-polarized photons, we get:

$$I^{\rm A}_{\rm K} \propto \frac{v_1^2}{\Delta_{\rm A}} \left[c_\lambda^2 (n_{{\rm C},\uparrow} - n_{{\rm C}',\downarrow}) - s_\lambda^2 (n_{{\rm C}',\uparrow} - n_{{\rm C},\downarrow}) + (c_\lambda^2 - s_\lambda^2)(n_{{\rm V},\uparrow} - n_{{\rm V},\downarrow}) \right], \tag{47}$$

$$I^{\rm B}_{\rm K} \propto \frac{v_1^2}{\Delta_{\rm B}} \left[s_\lambda^2 (n_{{\rm C},\uparrow} - n_{{\rm C}',\downarrow}) + c_\lambda^2 (n_{{\rm C},\downarrow} - n_{{\rm C}',\uparrow}) \right], \tag{48}$$

$$I^{\rm C}_{\rm K} \propto -\frac{v_3^2}{\Delta_{\rm C}} c_\lambda^2 (n_{{\rm V},\uparrow} - n_{{\rm V},\downarrow}), \tag{49}$$

where $c_\lambda^2 = (1+\tilde{\lambda})/2$ and $s_\lambda^2 = (1-\tilde{\lambda})/2$.

---

* emmanuele.cappelluti@ism.cnr.it
† hr745@bath.ac.uk
‡ federico.cilento@elettra.eu

[1] Liu, Gui-Bin and Shan, Wen-Yu and Yao, Yugui and Yao, Wang and Xiao, Di, Phys. Rev. B **88**, 085433 (2013).
[2] Rostami, H., Cilento, F. and Cappelluti, E., Nanomater. **14**, 707 (2024).
[3] Lin, K.-Q. et al., , Nat. Comm. **12**, 5500 (2021).
[4] Lin, K.-Q., Ziegler, J.D., Semina, M.A., Mamedov, V.J., Watanabe, K., Taniguchi, T., Bange, S., Chernikov, A., Glazov, M.M. and Lupton, J.M., Nat. Comm. **13** (2022).
[5] Cappelluti, E., Roldán, R., Silva-Guillén, J.A., Ordejón, P. and Guinea, F., Phys. Rev. B **88**, 075409 (2013).
[6] Gong, Z., Liu, G.-B., Yu, H., Xiao, D., Cui, X., Xu, X. and Yao, W., Nat. Comm. **4**, 2053 (2013).
[7] Liu, G.-B., Xiao, D., Yao, Y., Xu, X. and Yao, W., Chem. Soc. Rev. **44**, 2643 (2015).
[8] Kormányos, A. and Burkard, G. and Gmitra, M. and Fabian, J. and Zólyomi, V. and Drummond, N.D. and Fal'ko, V., 2D Mater. **2**, 022001 (2015).
[9] Xiao, D., Chang, M.-C. and Niu, Q., Rev. Mod. Phys. **82**, 1959 (2010).

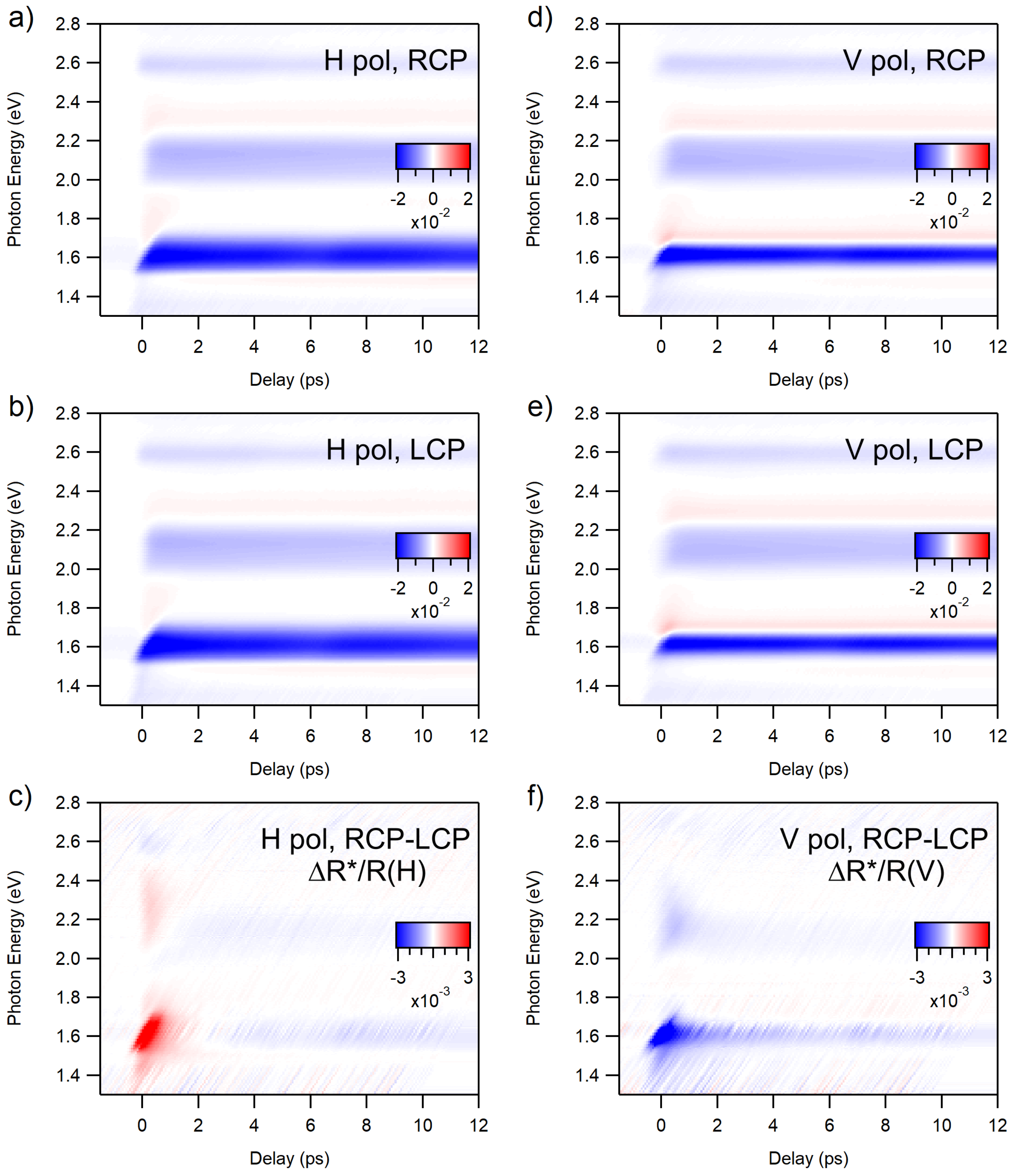

FIG. 1. Differential reflectivity maps $\Delta R/R$ for bulk 2H-WSe$_2$ with pump photon energy at $\hbar\omega_{\rm pump}$ = 1.55 eV. Panels (a)-(b) show $\Delta R/R$ along the H direction for RCP and LCP, respectively, while panel (c) shows the dichroism $\Delta R^*/R(= \Delta R/R_{\rm RCP} - \Delta R/R_{\rm LCP}$. Panels (d)-(f): as in panel (a)-(c) but along the V direction. The remarkable dichroism in both the H and V directions points out the evidence of a relevant Kerr rotation.

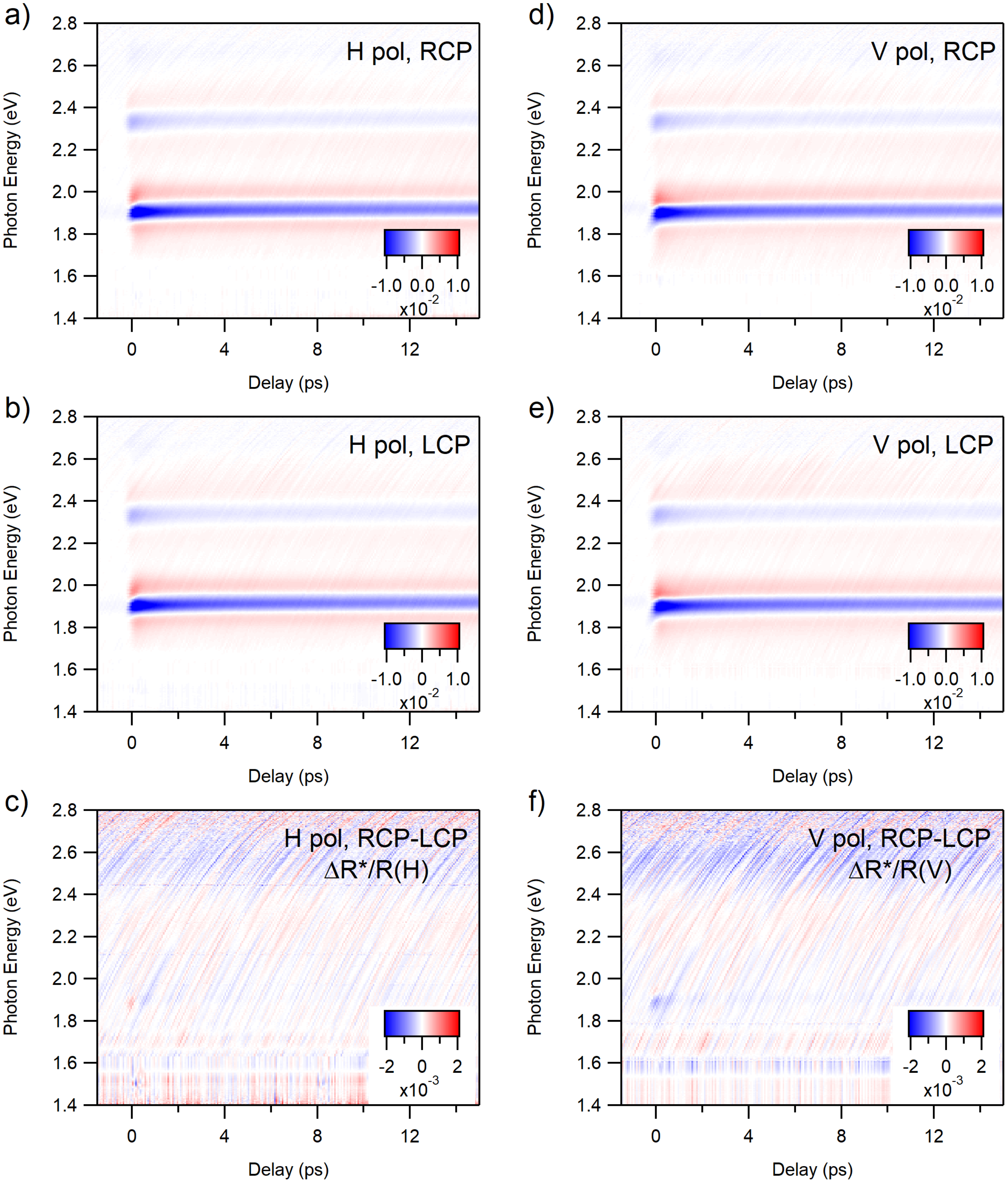

FIG. 2. Differential reflectivity maps $\Delta R/R$ for bulk 2H-WS_2 with pump photon energy at $\hbar\omega_{\mathrm{pump}} = 1.55$ eV. Panels (a)-(b) show $\Delta R/R$ along the H direction for RCP and LCP, respectively, while panel (c) shows the dichroism $\Delta R^*/R = \Delta R/R_{\mathrm{RCP}} - \Delta R/R_{\mathrm{LCP}}$. Panels (d)-(f): as in panel (a)-(c) but along the V direction. The lack of dichroism in both the H and V directions point out the absence of Kerr rotation under these circumstances.

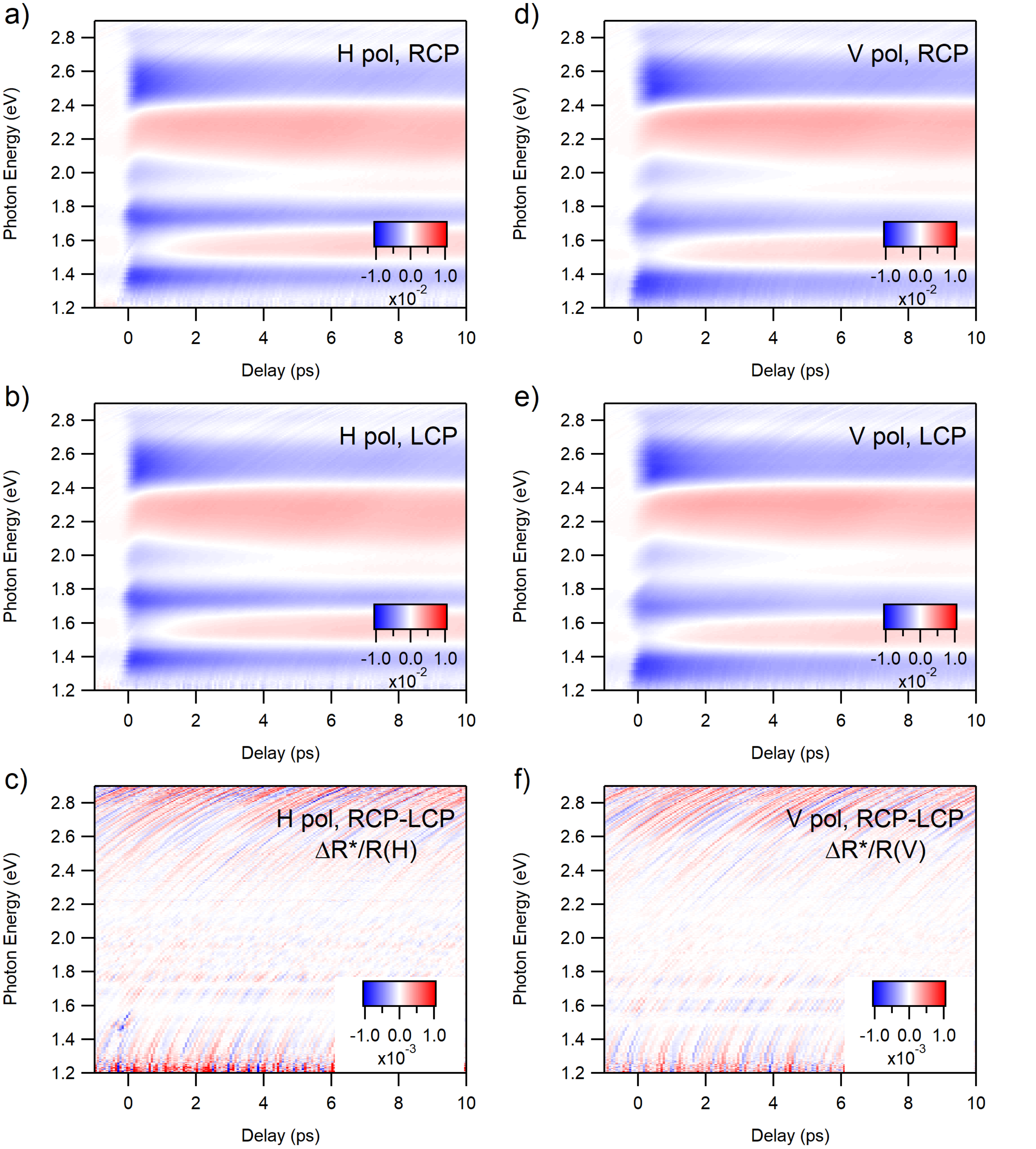

FIG. 3. Differential reflectivity maps $\Delta R/R$ for bulk 2H-MoTe$_2$ with pump phonon energy at $\hbar\omega_{\mathrm{pump}} = 1.55$ eV. Panels (a)-(b) show $\Delta R/R$ along the H direction for RCP and LCP, respectively, while panel (c) shows the dichroism $\Delta R^*/R = \Delta R/R_{\mathrm{RCP}} - \Delta R/R_{\mathrm{LCP}}$. The lack of dichroism in both the H and V directions point out the absence of Kerr rotation under these circumstances.
